# Human Surfactant Protein D Alters Oxidative Stress and HMGA1 Expression to Induce p53 Apoptotic Pathway in Eosinophil Leukemic Cell Line 

**DOI:** 10.1371/journal.pone.0085046

**Published:** 2013-12-31

**Authors:** Lakshna Mahajan, Hrishikesh Pandit, Taruna Madan, Poonam Gautam, Ajit K. Yadav, Himangi Warke, Curam S. Sundaram, Ravi Sirdeshmukh, P. Usha Sarma, Uday Kishore, Avadhesha Surolia

**Affiliations:** 1 Molecular Biochemistry and Diagnostics, Institute of Genomics and Integrative Biology (IGIB), New Delhi, Delhi, India; 2 Centre for Molecular Medicine, National Institute of Immunology (NII), New Delhi, India; 3 Innate Immunity, National Institute for Research in Reproductive Health (NIRRH), Mumbai, Maharashtra, India; 4 Proteomics Research Facility, Centre for Cellular and Molecular Biology (CCMB), Hyderabad, Andhra Pradesh, India; 5 Department of Obstetrics and Gynecology, K E M Hospital and Seth G. S. Medical College, Mumbai, Maharashtra, India; 6 Centre for Infection, Immunity and Disease Mechanisms (CIIDM), Brunel University, London, United Kingdom; Oxford University, United Kingdom

## Abstract

Surfactant protein D (SP-D), an innate immune molecule, has an indispensable role in host defense and regulation of inflammation. Immune related functions regulated by SP-D include agglutination of pathogens, phagocytosis, oxidative burst, antigen presentation, T lymphocyte proliferation, cytokine secretion, induction of apoptosis and clearance of apoptotic cells. The present study unravels a novel ability of SP-D to reduce the viability of leukemic cells (eosinophilic leukemic cell line, AML14.3D10; acute myeloid leukemia cell line, THP-1; acute lymphoid leukemia cell lines, Jurkat, Raji; and human breast epithelial cell line, MCF-7), and explains the underlying mechanisms. SP-D and a recombinant fragment of human SP-D (rhSP-D) induced G2/M phase cell cycle arrest, and dose and time-dependent apoptosis in the AML14.3D10 eosinophilic leukemia cell line. Levels of various apoptotic markers viz. activated p53, cleaved caspase-9 and PARP, along with G2/M checkpoints (p21 and Tyr15 phosphorylation of cdc2) showed significant increase in these cells. We further attempted to elucidate the underlying mechanisms of rhSP-D induced apoptosis using proteomic analysis. This approach identified large scale molecular changes initiated by SP-D in a human cell for the first time. Among others, the proteomics analysis highlighted a decreased expression of survival related proteins such as HMGA1, overexpression of proteins to protect the cells from oxidative burst, while a drastic decrease in mitochondrial antioxidant defense system. rhSP-D mediated enhanced oxidative burst in AML14.3D10 cells was confirmed, while antioxidant, N-acetyl-L-cysteine, abrogated the rhSP-D induced apoptosis. The rhSP-D mediated reduced viability was specific to the cancer cell lines and viability of human PBMCs from healthy controls was not affected. The study suggests involvement of SP-D in host’s immunosurveillance and therapeutic potential of rhSP-D in the eosinophilic leukemia and cancers of other origins.

## Introduction

Recent studies show that particular immune cell types, effector molecules, and pathways collectively form a functional cancer immunosurveillance process that detects and eliminates developing tumors [1]. The present study reports for the first time, another secreted pattern recognition molecule of innate immune system, Surfactant protein D (SP-D) that exerts antileukemic properties. SP-D, a member of collectin family, is composed of N-terminal collagen region and C-terminal C-type lectin domain or carbohydrate recognition domain (CRD) region [2]. It appears to perform a crucial role in linking innate and adaptive immunity [3]. Although initially discovered from the lung where it is secreted by type II and Clara cells [4], extra-pulmonary existence of SP-D has also been reported [5]. It also has been proposed to be a useful biomarker in certain carcinomas [6,7] and a range of lung-associated diseases [7,8]. Involvement of SP-D in immunosurveillance and immunomodulation is well documented in pulmonary allergy and asthma. Increasing the levels of SP-D in murine models of allergy has been reported to regulate the immune cell activation, pulmonary homeostasis and resistance to allergenic challenge [5,9]. Exogenous administration of full-length SP-D or rhSP-D has shown therapeutic effects in the hyper-eosinophilic SP-D gene deficient mice [10]. Previously, we reported that rhSP-D binds to human eosinophils and selectively induces apoptosis, oxidative burst and CD69 expression in the sensitised eosinophils isolated from allergic patients while eosinophils from healthy donors showed no significant change [8]. Furthermore, eosinophils from healthy donors, when primed with IL-5, exhibited an increase in apoptosis following incubation with SP-D suggesting that the healthy eosinophils in the absence of priming or activation do not undergo SP-D induced apoptosis [8].

The AML14.3D10 cell line exhibits advanced eosinophilic differentiation and is an outcome of autocrine activation of the intracellular cytokine (IL-3/GM-CSF/IL-5) signaling pathways by the endogenous GM-CSF production that also promote the cell line proliferation [11,12]. In view of the immunomodulatory properties of SP-D and its ability to selectively induce apoptosis in the primed eosinophils, we investigated the interaction of SP-D with the AML14.3D10 cell line. 

Here, we report that the native and recombinant version of full-length human SP-D, and rhSP-D (a recombinant homotrimeric fragment of human SP-D) showed anti-leukemic properties. There was a direct, dose, calcium and time dependent interaction of rhSP-D with the AML14.3D10 cell line. Treatment of the AML14.3D10 cells with rhSP-D resulted in G2/M arrest that led to the induction of apoptosis. Proteomic analysis revealed that rhSP-D treatment resulted in differentially expressed proteins that belonged to the following major functional categories: oxidoreductases and chaperones (cell stress); inflammation and survival; ubiquitin proteasome pathways; energy metabolism; transcription and translation; RNA binding and metabolism; cytoskeleton; vesicle fusion; synthesis and trafficking; metabolic enzymes and others. rhSP-D resulted in enhanced oxidative burst and antioxidant N-acetyl-L-cysteine (NAC) abrogated rhSP-D mediated apoptosis in the cell line. The study showed a significant reduction in the viability of acute myeloid leukemia cell lines (AML14.3D10 cell and THP-1 cell line), acute lymphoid leukemia cell lines (Jurkat and Raji) and human breast epithelial cell line (MCF-7), but unaltered viability of human PBMCs from the healthy controls in presence of rhSP-D. This suggests a possible role of SP-D in the immune surveillance against the leukemic cells.

## Materials and Methods

### AML14.3D10 and other leukemia cell lines

The AML14.3D10 cell line was provided by Dr. Michael Baumann and Dr. Cassandra Paul, Wright State University, Dayton, Ohio [11,12]. The cell line exhibited advanced eosinophilic differentiation and was grown in RPMI-1640 (Sigma) containing 10% fetal calf serum (FCS, Gibco BRL), 50μM 2-mercaptoethanol (Sigma), 1mM sodium pyruvate (Sigma), and Gentamicin (50μg/ml) (Gibco BRL) [11,12].

The leukemia cells lines such as THP-1: acute monocytic leukemia cell line, Jurkat: T lymphocyte acute cell leukemia cell line, Raji: Burkitt’s B-cell lymphoma and a breast cancer cell line of epithelial origin (MCF-7) were obtained from ATCC and grown under standard culture conditions.

### SP-D proteins

Native human SP-D was purified from the human amniotic fluid, obtained from pregnant women at term undergoing caesarean section at K E M Hospital and Seth G. S. Medical College, Mumbai, who gave written informed consent (approval from the ethics committees of NIRRH and K E M Hospital and Seth G. S. Medical College), using procedures described previously [13]. The protein preparation was found to be free from IgG, IgM, and IgE contamination by ELISA using the anti-human IgG, anti-human IgM, and anti-human IgE peroxidase conjugates, respectively. The amount of endotoxin was found to be 14pg/μg of SP-D. rhSP-D, composed of 8 gly-x-y repeats, homotrimeric neck and CRD region, was expressed under the bacteriophage T7 promoter in *Escherichia coli* as described earlier [14]. Its identity was confirmed by N-terminal sequencing, and was judged to be pure by SDS-PAGE and immunoblotting. The amount of endotoxin present in the preparations was estimated to be 4pg/μg of rhSP-D. A recombinant full length SP-D was isolated as previously described from supernatant of Chinese hamster ovary cells stably transfected with a cDNA clone of full-length rat SP-D [15]. Recombinant full length SP-D preparation contained <0.25pg endotoxin/μg protein. 

### Viability of AML14.3D10 cells and cell-cycle analysis

To study rhSP-D induced apoptosis and cell death, following assays were employed. Annexin V-FITC allowed the direct evaluation of cells (Phosphatidylserine externalization) in the very early stages in apoptosis before any nuclear changes occurred; propidium iodide was added to distinguish membrane permeabilized and late apoptotic cells [16], hypotonic propidium iodide assay was used to study nuclear changes (DNA fragmentation), where the appearance of sub-diploid DNA peak is a specific marker of apoptosis [17]. Further, to quantitate cell viability, MTT and trypan blue assays were performed. 

For the assays, 2 X 104 cells/100µl were plated in the 96-well microtiter plates in the presence of rhSP-D (1, 2.5, 5, 10 or 20µg/ml) or recombinant full length SP-D (10µg/ml) or native human SP-D (10µg/ml) or with the culture medium alone as experimental control and incubated for different time intervals (24, 48 and 72h). For MTT assay, 10µl MTT (5mg/ml stock) was added to each well and incubated for 4h. This was followed by addition of 100µl of 0.04N HCl in isopropanol and the absorbance was measured at 570nm [18]. In all cases, rhSP-D or recombinant full length SP-D or native human SP-D, was also added to wells containing medium alone to negate any possible interference with the colorimetric MTT assay. For studying the effect of rhSP-D mediated intracellular oxidative burst on cell viability, the cells were incubated with NAC (15mM) for 30 minutes followed by addition of rhSP-D (10µg/ml) for 24h and 72h. For MTT assay in this case, the cells were firstly rinsed with fresh media. The growth medium was then replaced with media without NAC followed by Modified MTT assay protocol as above.

The viability of AML14.3D10 cells was also determined by trypan blue exclusion assay following 24, 48 and 72h of incubation. The viable cell count was determined in five different optical fields with at least 300 cells each. 

For Annexin-V immunostaining assay, the protocol of Annexin V-FITC apoptosis detection kit (Calbiochem) was followed and the cells were immediately analyzed by flow cytometry. 

To analyze the cell cycle, and the proportion of apoptotic cells displaying a hypo-diploid DNA peak to that of viable cells, the method given Riccardi et al 2006 was followed [17]. For this, the cells fixed with 70% ethanol were resuspended in 1X PBS with 0.1% Triton X-100, 20µg/ml RNase and 70µg/ml propidium iodide and analyzed by flow cytometry. To demarcate the two populations of normal and apoptotic cells, IL-5 incubated AML14.3D10 cells were included, as IL-5 is known to maintain the viability of eosinophilic cells. The flow cytometry data was acquired on at least 20,000 cells from each sample on the Becton Dickinson FACS Calibur machine by the Cell Quest Pro software. For the cell cycle analysis, the Dean/Jett/Fox method of Flow Jo software was used. 

To investigate whether an initial incubation with rhSP-D is enough to achieve the induction of apoptosis, the effect of removal of rhSP-D after an initial treatment was evaluated. For this, the cells were incubated with rhSP-D (24h) and then washed with media to remove rhSP-D, resuspended in the media and further, incubated for 48h. The control cells were given equivalent washing and incubation. Another set of AML14.3D10 cells were treated with rhSP-D for 72h as a positive control for this experiment. 

The number of cells belonging to M region (apoptotic cells displaying a hypo-diploid DNA peak) divided by the total cell count was expressed as percentage cell apoptosis (% cell apoptosis). 

### Proteomics of rhSP-D treated AML14.3D10 cells for identification of differentially expressed proteins

To understand the molecular mechanisms involved in the induction of rhSP-D mediated apoptosis, we carried out proteomic analysis of AML14.3D10 cell line (3 × 10^5^ cells/ml), cultured in 10ml medium in 25cm^2^ tissue culture flasks, in the presence and absence of rhSP-D (10µg/ml) (5% CO_2_, 37°C). The cell line was treated with rhSP-D for 48h, a time period that would sufficiently prime the cells for a distinct pathway, before these cells extensively expressed proteins related to the apoptotic pathways. After harvesting, the cells were washed twice with cold salt free solution of Tris buffer sucrose (250mM sucrose, 10mM Tris buffer, pH 7.2) at 4°C. Total cell protein was extracted by sonication and interfering substances were removed by TCA/acetone preparation. The lyophilized sample was solubilized in the rehydration buffer (8M Urea, 2% CHAPS, DTT) with the protease inhibitor cocktail (Roche) and PMSF (Sigma) and estimated by the Bradford assay. Proteins (400µg) untreated or rhSP-D treated cells were separated in first dimension by the iso-electricfocusing on IPG strips, 17cm, pI range 5-8 (Biorad) on Protean isoelectric focusing (IEF) cell system (Biorad, Hercules, CA) and then in second dimension by SDS-PAGE on 12% Laemmli gels, following the manufacturers’ protocol. The gels were stained with reversible silver stain or colloidal coomassie blue G250 [19]. 2-DE gel images were acquired using Fluor-S MultiImager (Bio-Rad) and image analysis was carried out using PDQuest Image Analysis Software version 7.2 to identify protein spots showing differential expression in the treated AML14.3D10 cells. The images were taken under uniform settings for each experiment. For this, 3-4 major spots in different areas of the gel were used for fixing the coordinates. 2DE gels for rhSP-D treated and untreated cells were normalized for small variations in staining or protein loads using total optical density of the protein spots. Only those spots with 3-fold or more changes in expression intensity were selected for further MALDI-TOF-MS. The protein spots were picked from colloidal coomassie blue protein gels for the protein identifications by MALDI-TOF-MS. The colloidal coomassie blue protein staining showed a similar protein profile to that of silver stained gels and had sensitivity comparable to silver staining.

These selected proteins spots were excised and subjected to trypsin digestion. The tryptic digest was reconstituted in 0.6μl buffer (50% ACN/ 0.1% TFA) and analyzed using 4800 MALDI-TOF/TOF Proteomics Analyzer (Applied Biosystems) in positive ion reflector mode. The instrument was calibrated to <10ppm accuracy using calibration mixture of standard peptides in the mass range 800-4000m/z. MS data of the tryptic digests was acquired in an automatic mode. PMF (peptide mass fingerprinting) data was interrogated for protein identification with NCBI (National Center for Biotechnology Information) database (*Homo sapiens*) using the Mascot search program (Matrix Sciences). The analysis was done on global proteomic solutions (GPS) software (Applied Biosystems). The search parameters were partial methionine oxidation, no fixed modification, one missed cleavage and a mass tolerance of 100ppm, Mascot score ≥55, molecular mass unrestricted; sequence coverage > 10%. The MALDI-TOF analysis was repeated twice (from two gels) for all the samples followed by the search. 

For confirmation of protein identification by PMF, the peptides were further subjected to fragmentation by tandem MS (MS/MS). The MS/MS ion spectra of the peptides were interrogated with NCBI database (*Homo sapiens*) for confirmation of the protein IDs generated with PMF. A peptide showing individual ion score of ≥30 was considered as significant identification. The protein IDs that showed significant score after MS and/or MS/MS analysis were considered. The functions and functional category of proteins were assigned from the ExPASy database for biochemical pathways (http://www.expasy.ch/tools/pathways/).

### Detection of the expression levels of HMGA1 and apoptosis-associated factors by western blot

The cells were treated with rhSP-D, the total cell extracts were prepared in the ice-cold RIPA lysis buffer (Roche) and the resulting supernatants were assayed for protein content (BCA, Sigma). Equal amounts of the proteins (20-25μg) were separated using 10-12% SDS-PAGE. The protein bands were then transferred onto the nitrocellulose membranes and probed with the appropriate primary antibodies [HMGA1 (Santa Cruz Biotechnology Inc), phosphor p-53 (Santa Cruz Biotechnology Inc.), p21 (Cell Signaling Technology) cleaved caspase 7 (Cell Signaling Technology), caspase 9 (Cell Signaling Technology), cyclin B1 (Santa Cruz Biotechnology Inc.), cyclin D (Cell Signaling Technology), pcdc2 (Cell Signaling Technology) and cleaved PARP (Cell Signaling Technology). ECL (Pierce) detection system was used for immune detection. To show equal protein loading in each lane, the immune blotting was performed for β-actin (Abcam) or a non-specific protein band or its ponceau stain was demonstrated. To compare and quantitate the protein levels on blots, the ImageQuant TL v2005 was used.

### Intracellular oxidative burst and effect of NAC on rhSP-D-mediated AML14.3D10 apoptosis

Intracellular oxidative burst of the cells was determined using 2’, 7’-dichlorofluorescein diacetate (DCFHDA) by flow cytometry. For this, the treated cells were incubated with 5μM DCFHDA in the culture medium for 15 minutes at 37°C. The cells were thereafter washed twice with PBS, followed by immediate scanning by flowcytometry and collection of FL1 data. NAC was used for studying the effect of rhSP-D mediated intracellular oxidative burst on the apoptosis. For the assay, the cells were incubated with NAC (15mM) (Sigma) for 30 minutes followed by addition of rhSP-D (10µg/ml). The viability was measured by MTT assay and trypan blue staining, at 24 and 72h as described above.

### AML14.3D10 cell interaction with rhSP-D

To evaluate the involvement of CRD of SP-D in interaction with AML14.3D10 cells, the effect of calcium on rhSP-D (1, 5 and 10µg/ml) binding with AML14.3D10 cells was analyzed in presence of 2mM CaCl_2_ and/or 10mM EDTA using FITC labeled rhSP-D, as described earlier [8]. Briefly, rhSP-D was dialyzed extensively against FITC-labeling buffer (0.05M boric acid and 0.2M sodium chloride, pH 9.2), followed by incubation with FITC (20µl of 5mg/ml in dimethyl sulfoxide for each milligram of protein) for 2h at room temperature. The labeled proteins were dialyzed against buffer (0.1M Tris–HCl, pH 7.4, 0.1% w/v sodium azide and 0.2M NaCl) and stored at 4°C. The flurochrome to protein ratio of 5–6:1 was considered optimum for flow cytometric (FACS) studies. To evaluate any alterations in functional activities of rhSP-D and SP-D induced by FITC labeling, the unlabeled and FITC-labeled proteins were compared for their binding to *A. fumigatus* conidia and for the increase in fungicidal activity of neutrophils and alveolar macrophages, as reported earlier [8]. No significant difference was observed in the binding to conidia or fungicidal activity of FITC-labeled and -unlabeled rhSP-D and SP-D.

For the binding, AML14.3D10 cells were incubated with increasing concentrations of FITC-labeled rhSP-D for 45 minutes at 4°C in the staining buffer containing 2mM Ca^2+^ or 2mM Ca^2+^ and 10mM EDTA. The cells were washed and fixed for analysis by FACS. The binding was also analyzed in the presence or absence of maltose (100mM) using a polyclonal antibody against human SP-D raised in rabbit (Millipore) (1:100) followed by an anti-rabbit IgG-FITC (Invitrogen). FACS analysis was performed on at least 5,000 cells using FACScanTM (Becton Dickinson). 

To validate the involvement of CRD in the interaction, binding of rhSP-D (10µg/ml) to the AML14.3D10 cells was analyzed in presence of monoclonal antibody against the CRD of recombinant full length SP-D (Abcam) (recognizes CRD of rhSP-D on western blot) or cellular debris. Both monoclonal antibody and cellular debris [20] are known to bind the CRD of SP-D and hence, are likely to inhibit the CRD mediated interaction of SP-D with AML14.3D10 cells. The cellular debris was prepared by repeated freezing thawing (5 times) of the human PBMCs [20]. The set of antibodies used are as mentioned above in the maltose inhibition assay. FACS analysis was performed on at least 5,000 cells using FACScanTM (Becton Dickinson). 

### rhSP-D treatment of human PBMCs and leukemia cell lines of different origin

To characterize the anti-leukemic property of SP-D, various leukemia cells lines such as THP-1, Jurkat, Raji and a breast cancer cell line of epithelial origin (MCF-7) were used. For human PBMCs, venous blood (5ml) was drawn from 5 healthy adults (termed as healthy controls) (age 21–42 years, mean 30.2 years; two females) in heparinized tubes with written consent for participation in the study. The study was approved by the Institutional Human Ethical Committee, Institute of Genomics and Integrative Biology (CSIR), and recommended guidelines were followed during sample collection. Diluted blood was subjected to Ficoll-density (HiMedia) centrifugation and peripheral blood mononuclear cells (PBMCs) were isolated from the buffy layer. Human PBMCs and the cell lines were treated with or without rhSP-D (10 and 20µg/ml) for 48h. The viability was then analyzed by MTT assay and Annexin V- immunostaining as described above.

### Statistical analysis

Statistics were calculated using GraphPad Prism™ (version 4.0 and 6.0 GraphPad Software). Data are expressed as Mean ± SE. Dose response relationships were evaluated using one-way ANOVA or two-way ANOVA, and post hoc comparisons between individual treatments were made using Tukey's test. Instances involving two comparisons were evaluated by using Student’s *t*-tests. A value of p<0.05 was considered statistically significant. 

## Results

### SP-D decreased viability and induced apoptosis in AML14.3D10 cells

**Figure 1:**
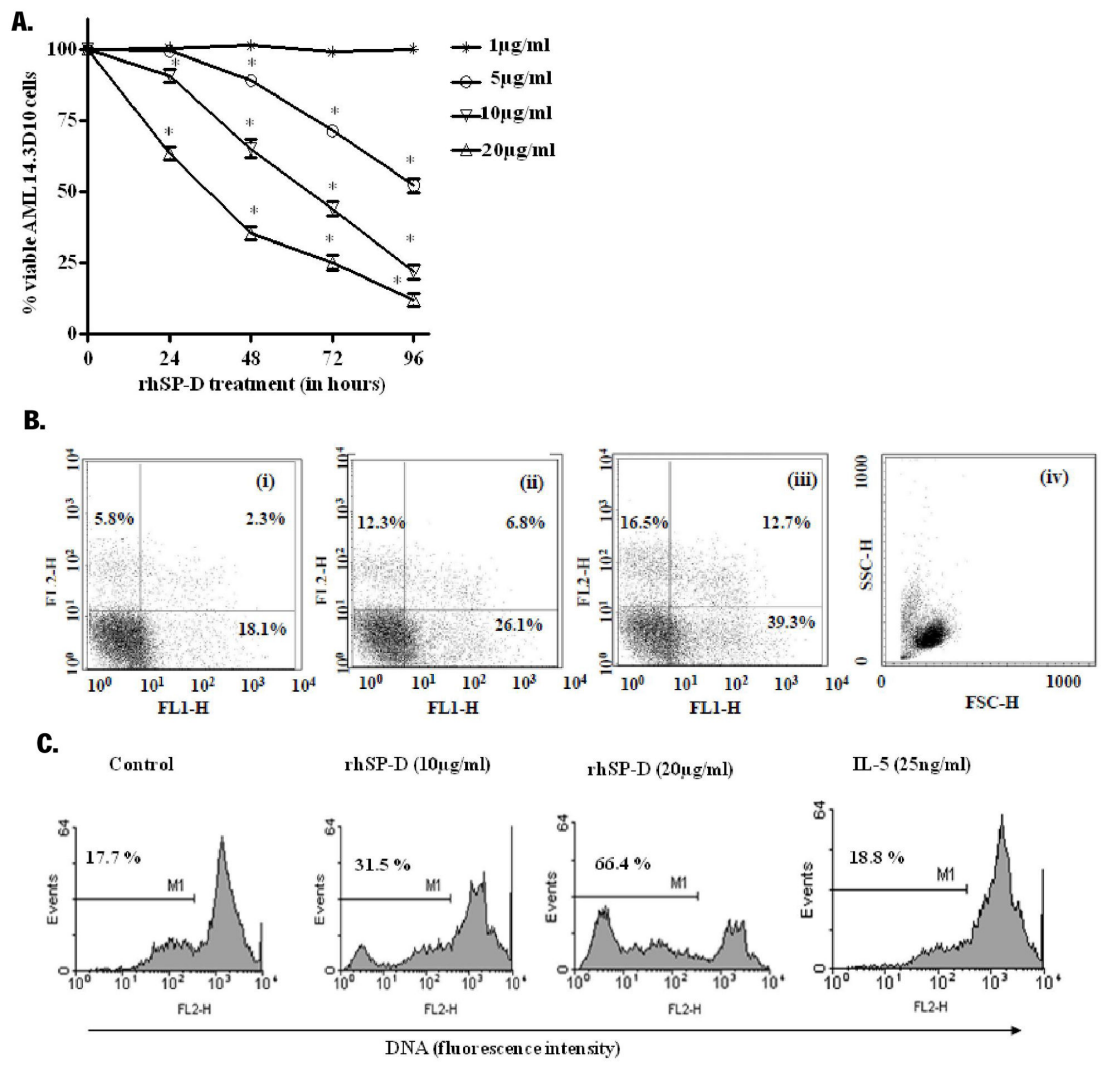
rhSP-D decreases viability of AML14.3D10 cells in a dose and time dependent manner. **A**. AML14.3D10 cells were incubated with varying concentrations of rhSP-D (1, 5, 10 or 20µg/ml) for the indicated time intervals. The viability of AML14.3D10 cells was assessed using a modified MTT assay. Results expressed as % viability ± SEM of cells, from 5 independent experiments, with 3 parallel samples in each experiment, in comparison to that of control (taken as 100%) at that time interval and compared by one-way ANOVA and Tukey’s test, * p<0.05 versus control. **B**. The cells were treated with rhSP-D (10 or 20µg/ml) for 48h and the apoptotic cells were detected by annexin-V-FITC/PI staining (**i**) Untreated, (ii) rhSP-D (10µg/ml), (iii) rhSP-D (20µg/ml), (iv) FSC and SSC. The increase in PI positive cells in rhSP-D treated cells represents late apoptotic cells. The figure shows representative scatter plots from one of the three independent experiments. **C**. Representative histograms for rhSP-D induced DNA fragmentation in AML14.3D10 cells studied by hypotonic propidium iodide staining. AML14.3D10 cells were incubated with rhSP-D (10 and 20µg/ml) for 72h at 37°C. IL-5 (25ng/ml) incubated cells with intact DNA were used to define marker (M1). The marker (M1) represents percentage apoptotic cells indicating the sub-diploid DNA. The figure shows representative histograms from one of the three independent experiments.

rhSP-D significantly decreased the viability of AML14.3D10 cells in a dose and time dependent manner, with faster kinetics at higher doses of rhSP-D (Figure 1A). Native SP-D and recombinant full length SP-D (10µg/ml) also significantly decreased the viability of the AML14.3D10 cells, as determined by MTT and Trypan blue dye exclusion assays (Table 1).

**Table 1:** Comparative effects of rhSP-D, recombinant full length SP-D and native human SP-D (10µg/ml) on the viability of AML14.3D10 cells.

	Percent viable cells		
**Time Interval (h)**	**rhSP-D**	**Recombinant full length SP-D**	**Native human SP-D**
24	89.3±3.2	91.5±2.1	90±2.6
48	63.9±4.3*	59±5.4*	75.4±3.6*
72	43±5.6*	50.2±4.8*	54.1±5.2*
Trypan blue dye exclusion assay (72h)	80±4.1*	79±2.6*	-

Values expressed as % viability in the treated cells as compared to that of control cells at different time intervals. Data are Mean ± SE from 5 independent experiments, with 3 parallel samples in each experiment and evaluated by using Student’s *t*-tests, * p<0.05.

A significant increase in the annexin-V positive cells was observed on treatment of AML14.3D10 cells with rhSP-D (10 and 20µg/ml) (Figure 1B). The recombinant full length SP-D at 10 and 20µg/ml also showed a significant increase of 19.56±3.2% and 52.17±4% in the annexin-V positive cells respectively at 48h. A significant increase (p<0.05) in the apoptotic cell count showing hypodiploid PI stained DNA in treatment with rhSP-D at 72h was observed (Figure 1C). 

Therefore, at 72h, when MTT showed viability of 43±5.6%, the cell membrane integrity was also lost to some extent and trypan blue staining demonstrated 80% viable cells i.e. cells with intact membrane integrity, while DNA fragmentation is observed in 31.5±4% cells as observed by hypotonic PI assay. The purified rhSP-D showed similar effect on the survival of AML14.3D10 cells to that of native and recombinant full length SP-D protein, the further studies were thus carried out using rhSP-D.

### rhSP-D resulted in accumulation of AML14.3D10 cells in G2 phase

**Figure 2:**
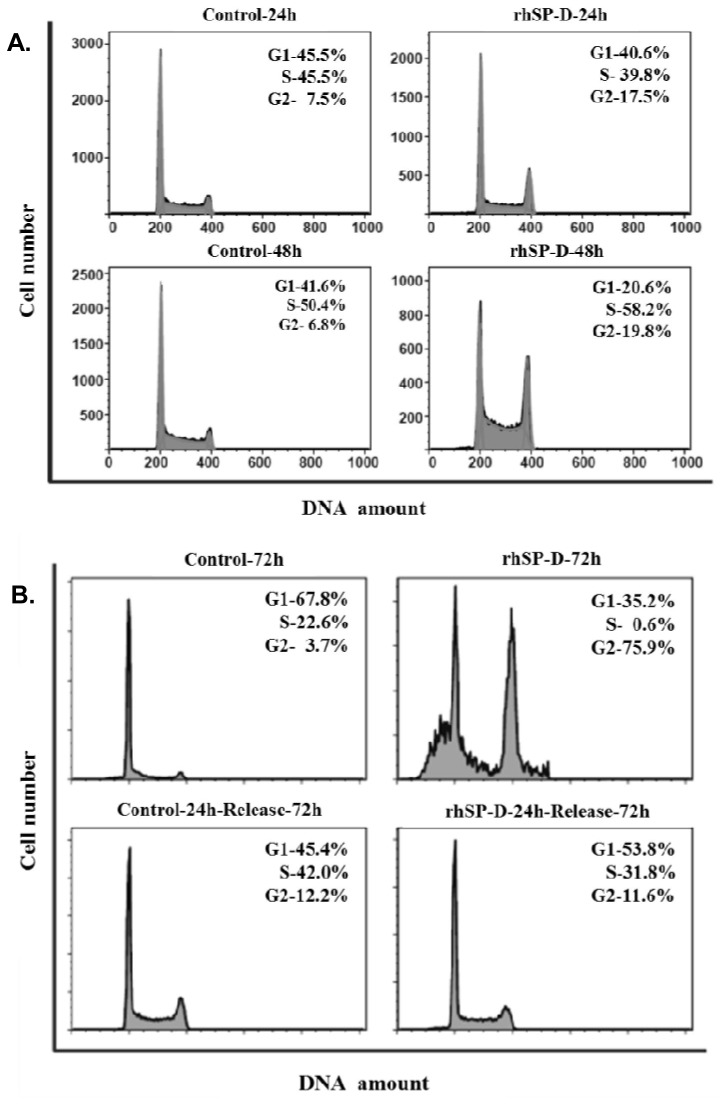
rhSP-D leads to G2/M phase arrest, while its removal post-24h reversed cell cycle arrest. **A**. AML14.3D10 cells were incubated in the presence of rhSP-D (10µg/ml) (Right panel) or in culture medium alone (control) (Left panel) for 24 or 48h, Top and Bottom panel, respectively. **B**. AML14.3D10 cells were incubated continuously with or without rhSP-D (10µg/ml) for 72h (rhSP-D-72h, Top right) and (Control-72h, Top left) respectively. Further, the cells were cultured in the presence of rhSP-D (10µg/ml) for 24h initially, followed by washing and removal of rhSP-D. The cells were then cultured in culture media for another 48h to complete an overall incubation of 72h (rhSP-D 24h-Release-72h, Bottom right); while the control cells were cultured without rhSP-D for 24h and then given same treatment as test (Control 24h-Release-72h, Bottom left).The cell cycle distribution was determined by flow cytometry of propidium iodide-stained DNA. The inset shows the percentage of total cells that are present in different phases. The flow cytometry data was acquired on Becton Dickinson FACS Calibur machine using Cell Quest Pro software. The figure shows representative histograms from one of the three independent experiments. The total population of cells has been divided in the G1, S and G2 phases based on the Dean/Jett/Fox method of FlowJo software.

Treatment of AML14.3D10 cells with rhSP-D (10µg/ml) for 24h and 48h resulted in a significant accumulation of cells in the G2 phase (Figure 2A). At 72h, rhSP-D resulted in a >20-fold increase in the G2 population from 3.7% in control cells to 75.9% in rhSP-D treated cells (Figure 2B, top left and right panel). There was also an increase in the sub G1 peak, suggesting that the treated cells had now fragmented DNA, a marker of cell apoptosis. Alternatively, the cells were incubated with rhSP-D for the first 24h, and subsequently rhSP-D was removed and cells maintained in the medium only for another 48h to complete an overall incubation of 72h (Figure 2B, bottom right panel). These cells showed a cell cycle distribution almost similar to the control untreated cells (Figure 2B, bottom left and right panel). The experiment unequivocally established that (a) rhSP-D induced a G2 block in AML14.3D10 cells, and (b) continuous presence of rhSP-D was required for sustained apoptosis, or removal of rhSP-D at 24h reversed the G2 block.

### rhSP-D treated AML14.3D10 cells showed differential expression of cell survival and redox-related proteins

**Figure 3:**
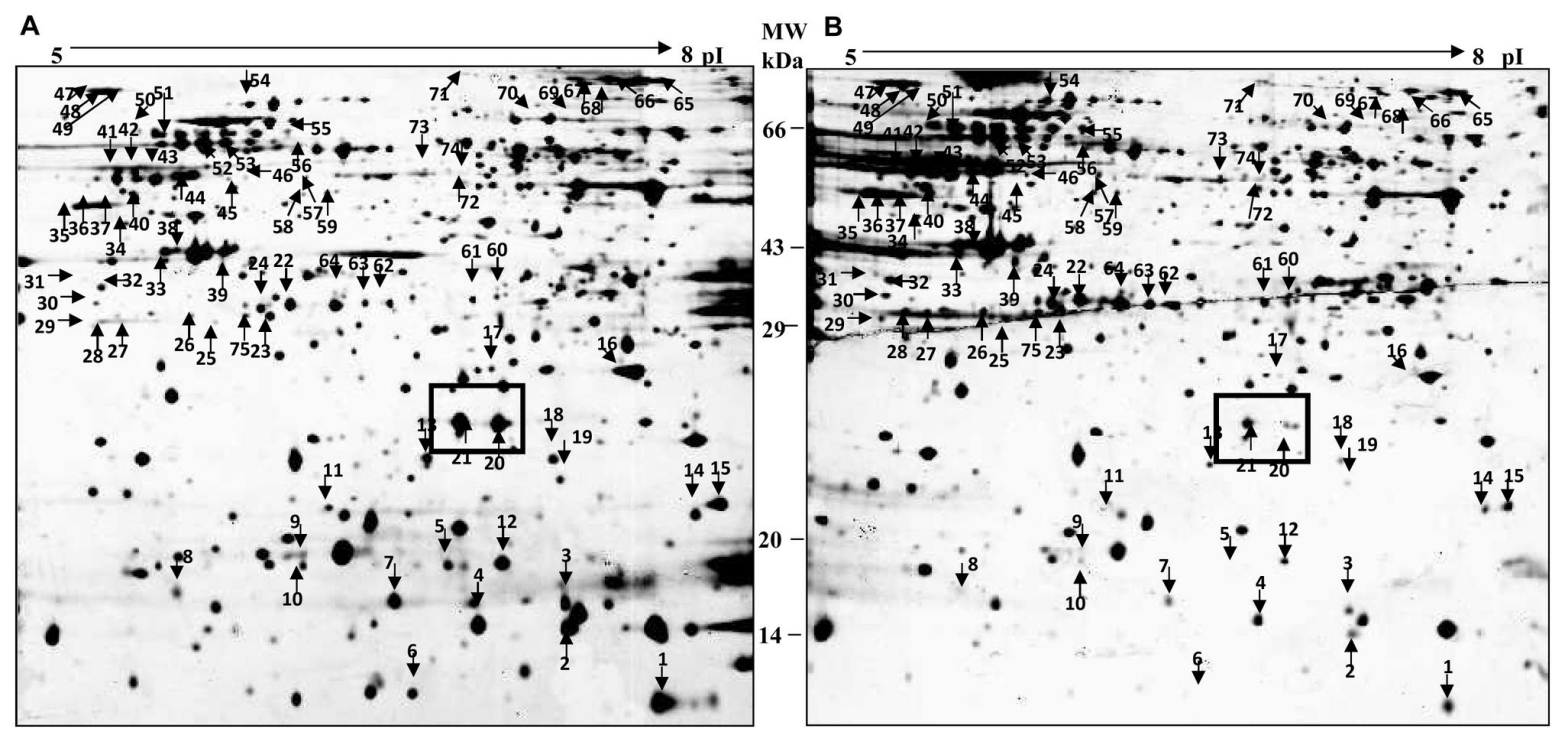
2-DE gel images of proteins extracted from AML14.3D10 cells. Proteins were extracted from AML14.3D10 cells **A**. untreated and **B**. treated with rhSP-D (10µg/ml) for 48h. Proteins (400µg) were separated by iso-electric focusing on IPG strips, 17cm, pI range 5-8 and SDS-PAGE on 12% Laemmli gels (n=3). Differentially expressed proteins (PDQuest Image Analysis Software version 7.2) showing a change of ≥ 3 were identified by MALDI-TOF-MS and MALDI-TOF-MS-MS (75 proteins, marked with arrows) (Table S1). The proteins marked in box are identified as HMGA1 proteins, which were downregulated on rhSP-D treatment.

Comparative analysis of silver stained 2DE-gels of rhSP-D (10µg/ml) treated and untreated AML14.3D10 cells for 48h showed a total of 134 proteins that exhibited a threefold- or more change in the expression. PMF analysis after MALDI-TOF MS and/or MALDI-TOF-TOF MS/MS analysis resulted in the identification of 75 out of 134 proteins (Figure 3, Table S1). These belonged to the following major functional categories: oxidoreductases and chaperones (cell stress related molecules); inflammation and survival; ubiquitin proteasome pathways; energy metabolism; transcription and translation; RNA binding and metabolism; cytoskeleton; vesicle fusion; synthesis and trafficking; metabolic enzymes and others. The differentially regulated proteins, their functional category, their role in normal cells and their significance in rhSP-D mediated apoptosis in eosinophilic cells are given in Table S2. The proteomic profile of rhSP-D treated AML14.3D10 cells provided us two important clues to rhSP-D mediated changes at the molecular level. Firstly, the treated cells showed increased expression of oxidoreductases and stress related molecules; and secondly, survival related proteins such as high-mobility group A1 (HMGA1) were found to be differentially expressed (Table 2). While an increased expression of cell stress related molecules was observed, the mitochondrial antioxidant defense system related proteins showed a drastic decrease (Table 2). 

**Table 2:** Differentially expressed proteins of AML14.3D10 in context to cell stress and cell survival, on rhSP-D treatment.

**Locus tag/ Functional category**	**Spot No.**	**Protein description**	**Biological Function**	**Increased/ Decreased**
**Cell Stress**				
gi|4759212	8	Tubulin-specific chaperone a	Tubulin complex assembly	Decreased
gi|8922762	9	Membrane-type 1 matrix metalloproteinase cytoplasmic tail binding protein-1	L-methionine salvage from methylthioadenosine (Oxidoreductase)	Decreased
gi|32483377	13	Peroxiredoxin 3 isoform b	Mitochondrion-specific peroxidase	Decreased
gi|7546411	14	Chain A, X-Ray Crystal Structure For Human Manganese Superoxide Dismutase, Q143a	Mitochondrial matrix superoxide dismutase	Decreased
gi|45768728	18	Ubiquinol-cytochrome c reductase, Rieske iron-sulfur polypeptide 1	Mitochondrial respiratory chain complex III	Decreased
gi|113422970	25	PREDICTED: similar to Copper chaperone for SOD	Copper chaperone for superoxide dismutase (CCS)	Increased
gi|62088106	32	Thioredoxin-like 1	Oxidoreductase	Increased
gi|42794775	45	Thioredoxin domain containing 5 isoform 2	Thioredoxin domain containing protein	Increased
gi|386758	47	GRP78 precursor	Cytoprotective function; induced in Endoplasmic stress	Increased
gi|6470150	48	GRP78 precursor	Cytoprotective function; induced in Endoplasmic stress	Increased
gi|6470150	49	GRP78 precursor	Cytoprotective function; induced in Endoplasmic stress	Increased
gi|3154294/7	52	Chaperonin	Mitochondrial chaperone	Increased
gi|24234688	54	Heat shock 70kDa protein 9B precursor	Molecular Chaperone	Increased
gi|21040386	55	HSPA9 protein	Candidate tumor suppressor gene; control of cell proliferation; a chaperone	Increased
gi|112491363	71	Chain A, Crystal Structure Of The Alpha Subunit Of Human S- Adenosylmethionine Synthetase 2	Methionine Adenosyltransferase Activity	Increased
gi|5174529	72	Methionine adenosyltransferase II, alpha	Synthesis of S-adenosyl-methionine	Increased
**Survival and Inflammation related proteins**				
gi|1633186	2	Chain A, Pkci-1-Apo+zinc	Protein Kinase C Interacting (inhibitory)	Decreased
gi|123377	20	High-mobility group I (HMGA1a)	Cell survival protein, oncogenic phenotype	Decreased
gi|62088704	56	Heterogeneous nuclear ribonucleoprotein K isoform a variant	Cell cycle progression; induction of p53 genes	Increased
gi|118084547	60	Aryl hydrocarbon receptor interacting protein	Signal transducer activity	Increased
gi|4505587	74	Platelet-activating factor acetylhydrolase, isoform Ib, gamma subunit 29kDa	Lipid Metabolism	Decreased

Proteins belonging to the categories (i) cell stress and (ii) inflammation and survival related proteins that showed altered expression (≥ 3-fold change) on treatment with rhSP-D (10µg/ml) in AML14.3D10 cells ( n=3). Spot number relates to the protein spots marked on the 2-DE gel images of proteins extracted from rhSP-D treated and untreated AML14.3D10 cells (Figure 3).

We focused on validating the role of oxidative burst and HMGA1 (one of the important survival related proteins) in rhSP-D mediated reduced cell survival. 

### NAC inhibited the effect of rhSP-D on intracellular oxidative burst and viability of AML14.3D10 cells

**Figure 4:**
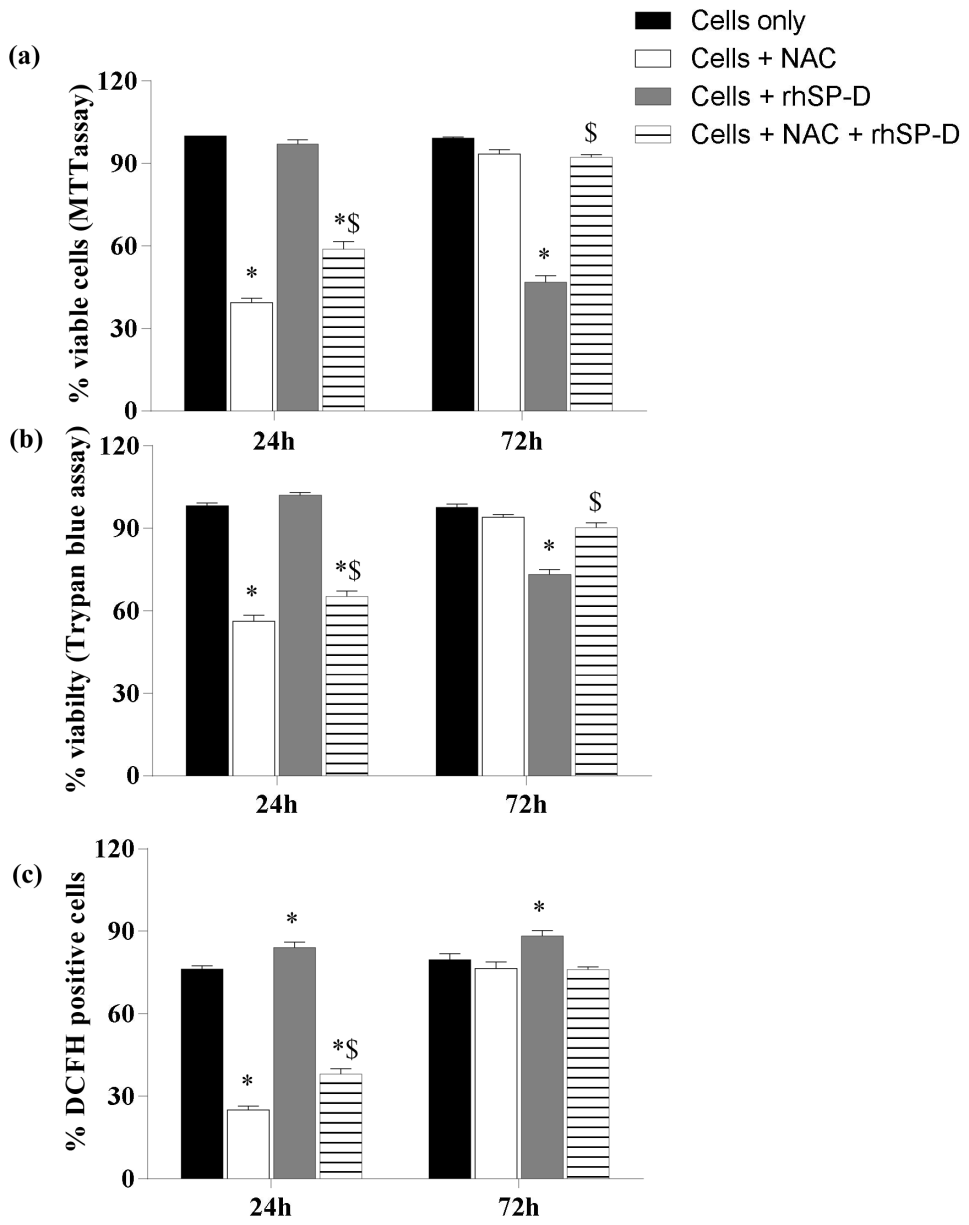
NAC decreases the effect of rhSP-D on viability and intracellular oxidative burst in AML14.3D10 cells. The cells were incubated with rhSP-D (10µg/ml) [rhSP-D], NAC (15 mM) [NAC] or pre-incubated with NAC for 30 minutes and then with rhSP-D [NAC + rhSP-D] for 24 and 72h. [Cells only] are untreated cells in culture media. The viability of the cells was determined by A. MTT assay and B. trypan blue exclusion assay. **C**. The intracellular release of H_2_O_2_ was determined by DCF by measuring FL1-H. % Viable cells in MTT assay [Cells only] taken as 100% for determining viability in treated cells. The data is Mean ± SE of three independent experiments and is compared by two-way ANOVA and Tukey’s multiple comparisons test* p<0.05 versus (Control) (cells cultured in medium alone), $ p<0.05 versus (Cells + rhSP-D).

At 24h, rhSP-D (10µg/ml) [rhSP-D] showed a significant increase in the oxidative burst (Figure 4C), however the cell viability was not affected as observed by MTT and trypan blue assay (Figure 4A and 4B). NAC decreased the oxidative burst as well as the viability of the AML14.3D10 cells alone [NAC] and of those treated with rhSP-D [NAC + rhSP-D] (Figure 4).

At 72h, rhSP-D [rhSP-D] treated cells showed a significant increase in the oxidative burst and a decrease in the viability (Figure 4). NAC treated cells [NAC] recovered from the antioxidant stress and showed the viability similar to control cells [Cells only]. Although NAC [NAC] had lost its effect on the oxidative burst and viability at 72h, the cells pre-incubated with NAC followed by rhSP-D treatment [NAC + rhSP-D] did not show any significant change in oxidative burst and the viability as compared to control cells [Cells only].

### rhSP-D downregulated HMGA1 expression and induced p53 dependent apoptosis

**Figure 5:**
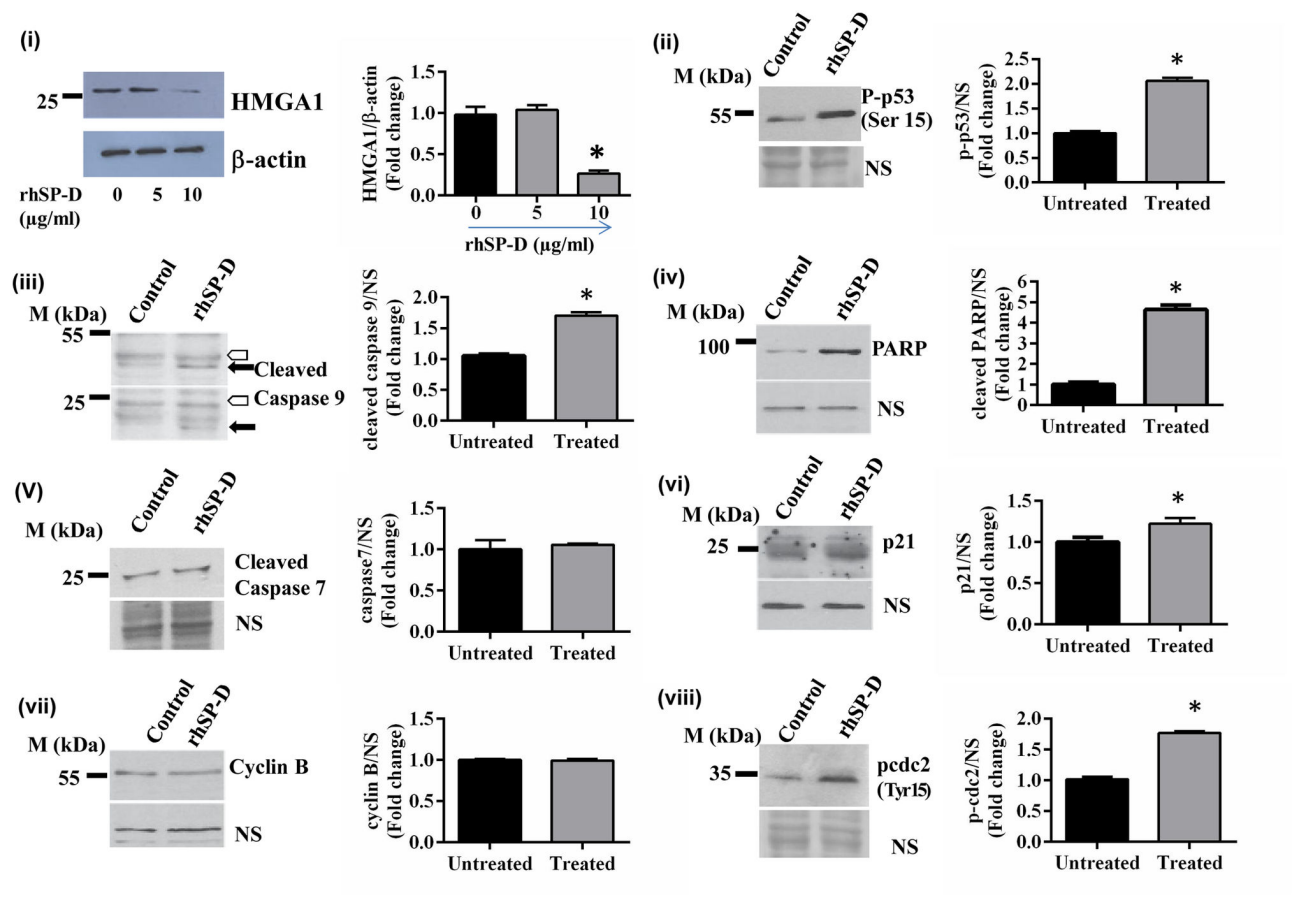
rhSP-D treatment results in downregulation of HMGA1 levels and activates p53 and the apoptotic pathway. (**i**) AML14.3D10 cells treated with rhSP-D (5 and 10µg/ml) for 48h, were analyzed for HMGA1 expression by western blot. The cells incubated with rhSP-D (10µg/ml) for 24h were also analyzed for the levels of (ii) phospho-p53 (Ser15), (iii) cleaved caspase 9, (iv) cleaved PARP, (**v**) cleaved caspase 7, (vi) p21, (vii) cyclin B and (viii) phospho-cdc2 by immunoblotting. NS points to a non-specific band that displays equal protein loading in different lanes. Cleavage of caspase 9 (iii) produces two bands of molecular mass 37 kDa (upper panel; black arrow) and 17 kDa (lower panel; black arrow). Hollow arrow points to a non-specific band that displays equal protein loading. On the right is shown the quantification of the normalized values, *p<0.05, n=3 independent experiments.

Downregulation of HMGA1 expression in the rhSP-D treated cells, as evident in the proteomics profile, was confirmed by western blot analysis. Expression of HMGA1 decreased in the presence of rhSP-D (10µg/ml) at 48h and was not altered significantly either at lower rhSP-D concentration (5µg/ml) (Figure 5(i)) or at an early time point i.e. at 24h. The activated p53 is a mediator of increased oxidative burst, G2/M phase block, and apoptosis. Thus, we analyzed levels of activated p53 in rhSP-D treated cells. 

Treatment of AML14.3D10 cells with rhSP-D (10µg/ml) for 24h led to increased phosphorylation of p53 at the Serine 15 position (Figure 5(ii)). rhSP-D treatment caused cleavage of caspase 9 (Figure 5(iii)) and PARP (Figure 5(iv)), the markers of apoptosis. The caspase-7 was not cleaved (Figure 5(v)). In addition, we found an increase in the expression of p21 levels (Figure 5(vi)) and a significant increase in the phosphorylation of cdc2 (Figure 5(viii)). Levels of cyclin B (Figure 5(vii)) were unaltered after 24h treatment with rhSP-D.

### rhSP-D showed calcium dependent-binding via its carbohydrate recognition domain

**Figure 6:**
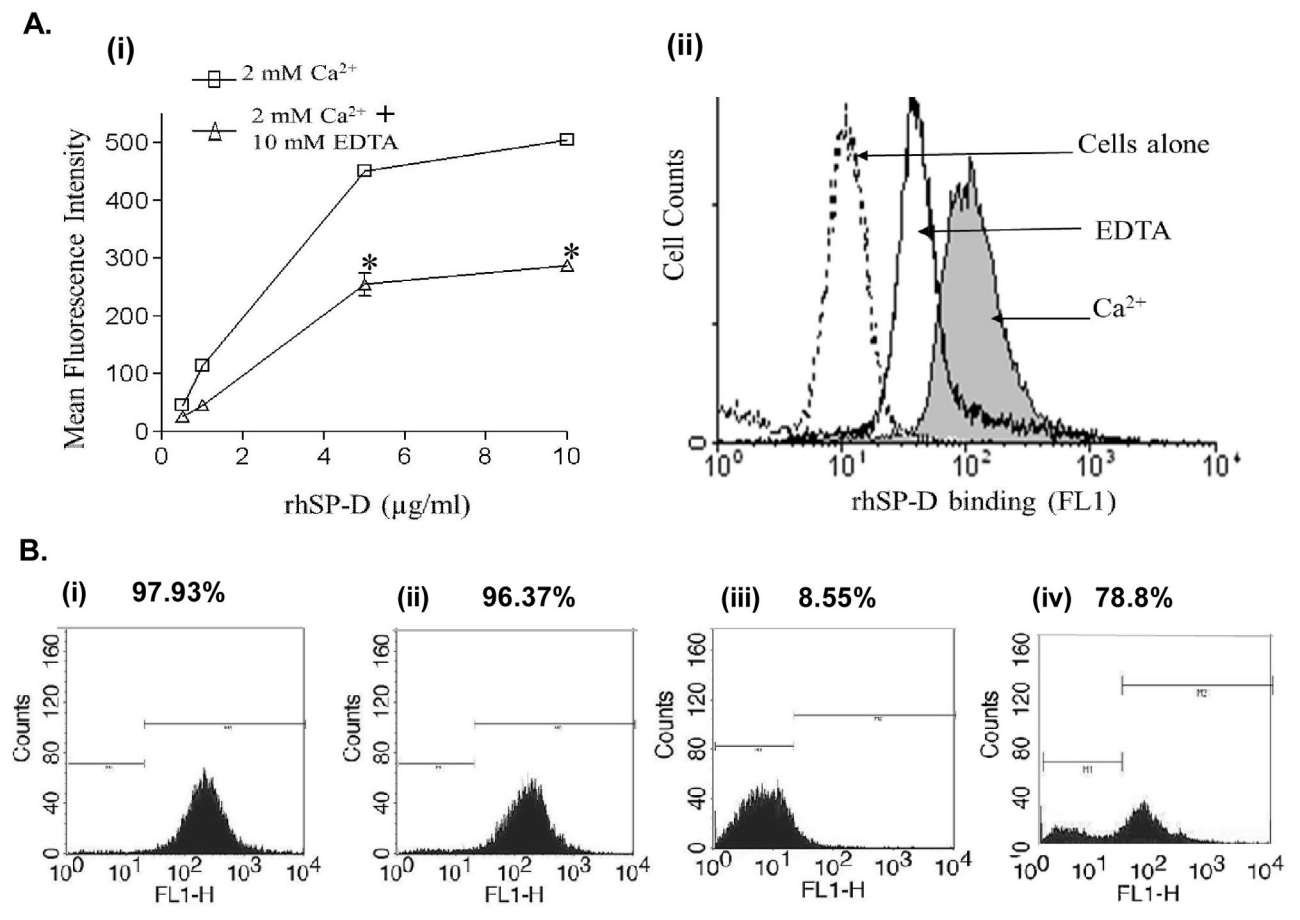
Involvement of CRD region of SP-D in its interaction with AML14.3D10 cells. **A**. **rhSP-D shows dose and calcium dependent binding to AML14.3D10 cells**. (**i**) **AML14.3D10** cells were incubated with increasing concentrations of the FITC-labeled rhSP-D in the presence of 2mM Ca^2+^ (Ca^2+^) or 2mM Ca^2+^ and 10mM EDTA (EDTA). Data are Mean ± SE of the mean fluorescence of rhSP-D binding to the AML14.3D10 cells; n=3 independent experiments. Significant difference from Ca^2+^ alone: * p<0.05 by Student’s t-test. (ii) A representative FACS histogram of AML14.3D10 cells with FITC-labeled rhSP-D (5µg/ml) showing decreased binding in presence of EDTA. **B**. **Engagement of CRD with cellular debris inhibits the interaction with AML14.3D10 cells**. AML14.3D10 cells were incubated with rhSP-D (10µg/ml) (**i**) or rhSP-D (10µg/ml) pre-incubated with monoclonal antibody against CRD domain of SP-D (ii) or rhSP-D (10µg/ml) in the presence of cellular debris (10 debris : 1 AML 14.3D10 cells) (iii) or rhSP-D (10µg/ml) in the presence of 100 mM Maltose (iv). The cells were then washed and probed with anti-native human SP-D raised in rabbit followed by incubation with anti-rabbit IgG-FITC. The cells were washed, and fixed and analyzed by FACS. % indicates the percentage of fluorescent AML14.3D10 cells. The figure shows representative histograms from one of the three independent experiments.

We have previously shown a direct interaction of rhSP-D with sensitized eosinophils [8]. Thus, it was likely that the modulation of cellular activities such as increase in oxidative burst and apoptosis in AML cells were also mediated by a direct interaction of rhSP-D with AML14.3D10 cells. FITC labeled rhSP-D showed a dose and calcium dependent binding to the AML14.3D10 cells (Figure 6A). Addition of EDTA (10mM) reduced the rhSP-D binding to AML14.3D10 cells to almost half at all rhSP-D concentration (Figure 6A). The calcium dependence and EDTA inhibition of the rhSP-D interaction with AML14.3D10 cells suggested the involvement of CRD. The interaction was further validated for CRD involvement by studying the binding of rhSP-D with AML14.3D10 cells in the presence of maltose or monoclonal antibody against the CRD or cellular debris.

The presence of maltose (100mM) (Figure 6B (iv)) however did not significantly reduce binding of rhSP-D (10µg/ml) to AML14.3D10 cells. The CRD recognizing MoAb did not bind to AML14.3D10 bound rhSP-D suggesting that the CRD region of rhSP-D was pre-occupied in binding to the cellular ligand (data not shown). We hypothesized that the pre-incubation of this MoAb with rhSP-D may competitively inhibit their interactions with the AML14.3D10 cells. However, MoAb did not alter interactions of rhSP-D (Figure 6B (ii)) with the AML14.3D10 cells. This suggested that affinity of the interaction of rhSP-D to AML14.3D10 cells was probably strong enough to displace the monoclonal antibody bound to rhSP-D. The cellular debris, known to interact with CRD of SP-D, almost completely inhibited the interaction of rhSP-D (10µg/ml) (Figure 6B (iii)) with the cells.

### rhSP-D did not alter viability of healthy PBMCs but reduced viability of various leukemia cell lines

**Figure 7:**
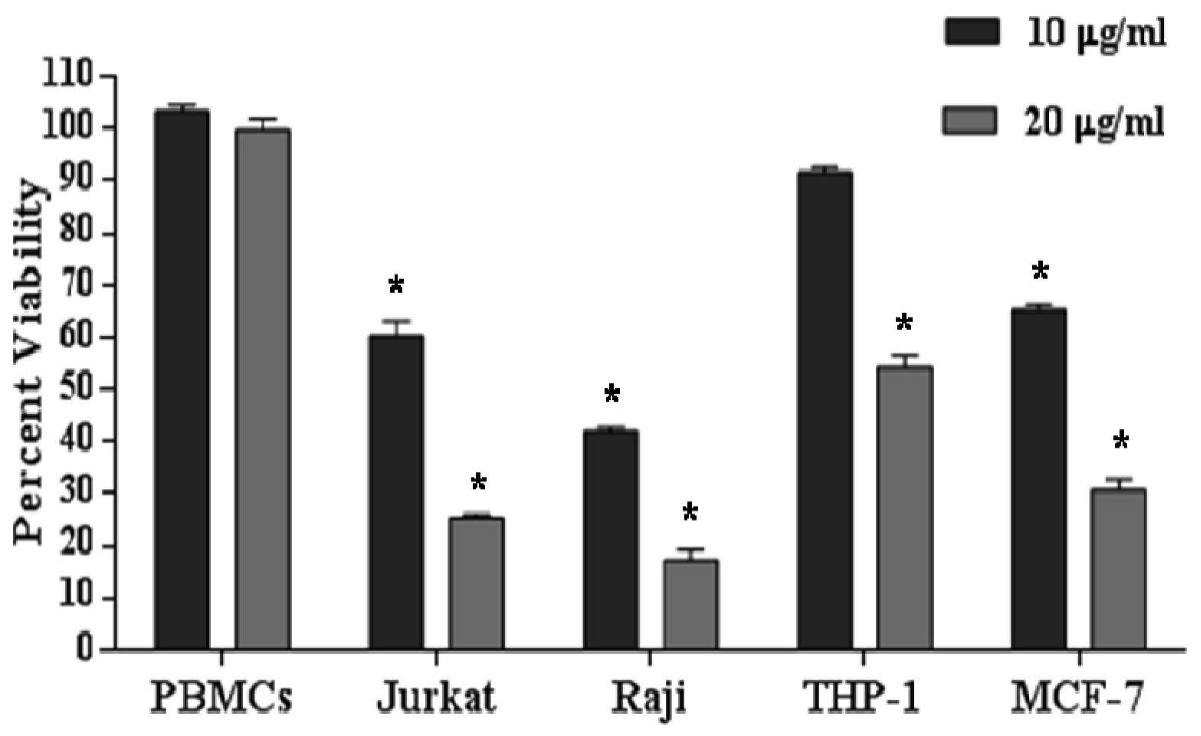
rhSP-D induces apoptosis in leukemia cell lines having different origins. Human PBMCs, AML14.3D10, Jurkat, Raji, THP-1 or MCF-7 cells were treated with rhSP-D (10 or 20µg/ml) for 48h. The viability of rhSP-D treated or untreated cells was assessed by a modified MTT assay. Results are expressed as % viability ± SEM in comparison to control from 3 independent experiments, and evaluated by Student’s t-tests, * p<0.05.

We found that rhSP-D treatment did not significantly affect the viability of PBMCs isolated from healthy donors at rhSP-D concentrations 10 and 20µg/ml as checked by MTT assay (Figure 7) and Annexin-V FITC staining (Figure S1). Further the effect of rhSP-D on various leukemia cells lines belonging to the category of acute myeloid leukemia (THP-1: acute monocytic leukemia cell line), acute lymphoid leukemia (Jurkat: T lymphocyte acute cell leukemia cell line, Raji: Burkitt’s B-cell lymphoma) and a breast cancer cell line of epithelial origin (MCF-7) were studied. rhSP-D (10µg/ml) resulted in a significant decrease in the viability of Jurkat (60.2±3.9%), Raji (42.0±1.1%) and MCF-7 cells (65.1±2%), while viability of THP-1 was not significantly altered (Figure 7, n=3). Increasing the concentration of rhSP-D to 20µg/ml further decreased the viability of all the cell lines (including THP-1) by two-fold suggesting a dose dependent effect (Figure 7). The study indicated that rhSP-D specifically exerted its apoptotic effect on the cancer cell lines.

## Discussion

We report, for the first time, the ability of rhSP-D to selectively reduce the survival of cancerous cells-AML14.3D10, Jurkat, Raji, MCF-7 and THP-1, while survival of healthy PBMCs was unaltered. In the presence of rhSP-D, AML14.3D10 eosinophilic leukemia cells showed a significant increase in the number of annexin-V positive cells, G2 phase and DNA fragmentation suggesting induction of G2/M arrest and apoptosis. Apoptosis is a sequential process of variable duration, depending on inducer and cell type [21]. The phosphatidylserine externalization (Annexin-V staining), followed by mitochondrial dysfunction (MTT assay) and the DNA fragmentation (hypotonic PI staining), followed by loss of membrane integrity (trypan blue staining), were the events observed during rhSP-D induced apoptosis in AML14.3D10 cell line. 

Proteomics profile showed differential expression of oxidative burst related proteins, among others, in rhSP-D treated cells. This suggested that probably, the localized production of ROS induced by rhSP-D was compensated by the increased expression of cellular reducing agents such as glutathione synthetase and thioredoxins (Trx) and co-ordinated induction of proteins with radical-scavenging properties and protective activities as different electrophoretic forms of heat shock protein 70 (HSP 70), chaperonins (mitochondrial chaperones), calreticulin precursor variant, copper chaperone for superoxide dismutase, etc [22,23]. We confirmed that rhSP-D significantly up-regulated intracellular oxidative burst in AML cells, while scavenging of ROS with NAC led to the inhibition of rhSP-D induced apoptosis of AML cells. These results implied that the increase in oxidative burst was likely to precede the induction of apoptosis in rhSP-D treated cells. Similar to rhSP-D, some of the natural products with anti-leukemic effects have been reported to enhance cytotoxicity by increasing the intracellular oxidative burst [24]. In contrast to above, the mitochondrial antioxidant defense system was compromised in rhSP-D treated cells. There was a decreased expression of Ubiquinol-cytochrome c reductase (complex III of the electron transport chain), Peroxiredoxin 3 isoform b and Mitochondrial matrix superoxide dismutase. Under such conditions with mitochondrial antioxidant defense mechanisms compromised, there is increased mitochondrial generation of reactive oxygen species, irreversible damage of mitochondrial DNA, membrane lipids and proteins, resulting in mitochondrial dysfunction. This may trigger the intrinsic pathway of apoptosis and ultimately cell death [25]. 

The proteomics approach identified large scale molecular changes initiated by SP-D in a human cell for the first time. Another important mechanism contributing to anti-leukemic activity of rhSP-D was reduced expression of survival related proteins such as HMGA1. HMGA1 is known to induce a transformed phenotype in cultured, hematopoietic cells and causes aggressive leukemia in transgenic mice [26]. Conversely, inhibiting HMGA1 expression is found to block phenotype transformation in a range of cancer cells [27].  HMGA1, an oncogenic transcription factor, is over-expressed in various high-grade malignancies, including AML (acute myeloid leukemia), ALL (acute lymphoid leukemia) and Burkitt’s lymphoma [28]. In view of this, we also found that the cell lines belonging to this category, AML (AML14.3D10 cell and THP-1 cell line), ALL (Jurkat and Raji) and human breast epithelial cell line (MCF-7) showed decreased viability in presence of rhSP-D.

 Increased levels of p53 phosphorylated at Ser15 in rhSP-D treated AML14.3D10 cells were plausibly mediated by oxidative stress [29] and downregulated HMGA1. The proteomics data also showed that on rhSP-D treatment heterogeneous nuclear ribonucleoprotein K (hnRNP K) was upregulated that is known to be induced by stress and is required for the induction of p53 target genes [30]. Increased levels of p21 were also observed in rhSP-D treated AML14.3D10 cells. Activated p53 and increased p21 expression cause the inactivation of cyclin B-cdc2 complexes that are pivotal in regulating G2/M transition and leads to either DNA repair or apoptosis [31,32]. The rhSP-D treated cells also demonstrated an increased Tyr15 phosphorylation of cdc2, suggesting activation block on the cdc2 [33]. Treatment of AML14.3D10 cells with rhSP-D resulted in the activation of caspase-9, the intrinsic pathway of apoptosis [34]. Once initiated, caspase-9 goes on to cleave procaspase-3 or procaspase-7 that in turn cleaves several cellular targets, including PARP, a well-known marker of apoptosis [35]. Although, PARP was found to be cleaved in the rhSP-D treated cells, cleavage of caspase-7 was not observed. This indirectly suggests that an intermediate activation of caspase-3 may be leading to the cleavage of PARP. 

**Figure 8:**
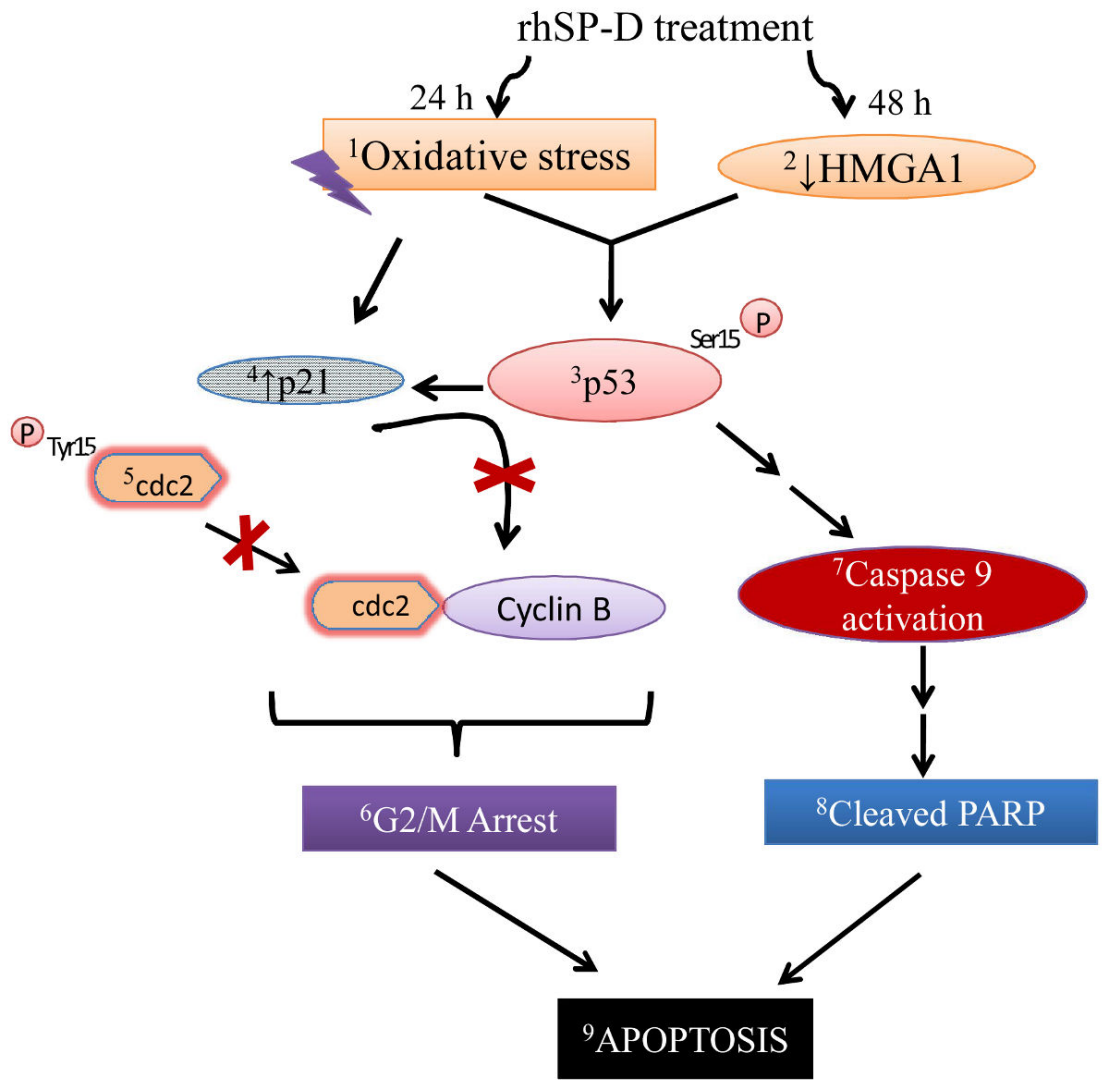
Proposed mechanism for rhSP-D mediated apoptosis in AML14.3D10 eosinophilic leukemic cells. rhSP-D leads to oxidative stress and reduction in the levels of HMGA1. This possibly results in phosphorylation of p53 (Ser15) and Cdc2 (Tyr15). p21 levels were also found to be upregulated. p21 elevation and Cdc2 phosphorylation contribute to Cdc2 inhibition leading to G2/M arrest. Taken together, these overall changes lead to G2/M arrest followed by apoptosis. The superscripts (1-9) are the changes observed during the study.

The proposed mechanism for the rhSP-D mediated apoptosis in AML14.3D10 eosinophilic leukemic cells has been depicted in Figure 8. At 24h, rhSP-D induced increased oxidative stress, p53 Ser15 phosphorylation, elevated p21 expression and inhibitory Tyr15 phosphorylation of cdc2 that would reduce the activity of Cdc2-cyclin B1 leading to G2/M arrest [36]. rhSP-D mediated reduction in HMGA1 level was observed at 48h which would further accumulate activated p53 and caspase 9 activation [37]. The removal of rhSP-D at 24h reversed the G2/M cell cycle arrest in unsynchronus population of AML14.3D10 cells suggesting that cells at a particular growth phase were affected and it required continuous presence of rhSP-D in the medium for the sustained induction of apoptosis, or the downstream events that involve decrease in HMGA1 levels might be required. The mechanism of rhSP-D induced apoptosis in other leukemia cell lines, however needs to be studied and confirmed.

rhSP-D primarily comprises of carbohydrate recognition domain (CRD), ability of rhSP-D to reduce cell survival and induce apoptosis similar to that of SP-D, suggested involvement of CRD in these functions. We found that interaction of SP-D with AML cells, as with a number of other immune cells e.g. macrophages, dendritic cells and neutrophils, could be through its CRD, and was enhanced in the presence of calcium and inhibited by EDTA [38]. However, maltose did not significantly affect rhSP-D binding to AML14.3D10 cells. Similar to our results, maltose was not able to inhibit the interaction of SP-D with T lymphocytes although interacting through CRD [39]. The inhibition of the interaction of SP-D with AML14.3D10 cells in presence of cell debris, further suggested that the collagen domain is not involved in the interaction and indirectly shows that the CRD may be involved [20]. 

We report the ability of rhSP-D to selectively reduce the survival of cancerous cells-AML14.3D10, Jurkat, Raji, MCF-7 and THP-1, while survival of healthy PBMCs was unaltered. We could not find the exact reason for this differential behavior, but such behavior of surfactant proteins is well documented. Role of SP-D has been established to create homeostasis in the allergy conditions [5,9,10]. We have also earlier found that rhSP-D induced apoptosis in cells in activation phase as in primed eosinophils while eosinophils from healthy individuals did not show any change [8]. A similar response has been reported earlier for another surfactant protein, SP-A, in regulating chemotaxis of inflammatory BAL neutrophils while peripheral neutrophils were unresponsive to SP-A towards known chemoattractants, depending on the activation state of the cells [40]. The leukemic cell lines are highly dividing and some have increased levels of HMGA1, while PBMCs including eosinophils, donot multiply and the HMGA1 protein levels are hardly detectable in normal adult tissue [41] and thus these are not dependent on the similar pathways for survival as leukemia cell lines. Further whilst NAC, an antioxidant, is known to inhibit spontaneous apoptosis in eosinophils [8]; it decreased the viability of AML14.3D10 eosinophilic cell line (24 h). This can be explained in view of earlier studies on tumor cells which report that NADPH oxidase mediated generation of ROS is required for survival of undifferentiated cells in human promyelocytic leukemia cell line HL-60 [42]. The ROS production has been observed in hematopoietic malignancies as in acute myeloid leukemias (AML) [42] and required for cell survival. rhSP-D resulted in an increase in oxidative burst in both the cell types. But on one hand, in IL-5 primed eosinophils, NAC could not affect rhSP-D mediated decrease in cell viability; in eosinophilic cell line NAC inhibited rhSP-D mediated apoptosis. In view of this, the anticancer agents have been found to enhance cytotoxicity by increasing the NADPH oxidase activity, similar to what we observed for SP-D [24]. 

In summary, SP-D acts as a potent inducer of apoptosis in the eosinophilic and other cancer cells and enhances the apoptotic cell uptake by macrophages [8]. This suggests that SP-D may be an integral component of human innate immune surveillance against cancer cells. The recombinant fragment of human SP-D, rhSP-D, may be an interesting and novel therapeutic strategy for such disorders. 

## Supporting Information

Figure S1 **rhSP-D does not affect viability of human PBMCs from healthy donors.**
(DOCX)

Table S1 **Identification of differentially expressed proteins of AML14.3D10 cell line on treatment with rhSP-D by MALDI-TOF and/or MALDI-TOF-MS/MS analysis.**
(DOCX)

Table S2 **Function and functional categories of differentially expressed proteins of AML14.3D10 cells on treatment with rhSP-D.**
(DOCX)
