# Supplementary material for: Human Surfactant Protein D Alters Oxidative Stress and HMGA1 Expression to Induce p53 Apoptotic Pathway in Eosinophil Leukemic Cell Line
Source: PLoS One. 2013 Dec 31;8(12):e85046. doi: 10.1371/journal.pone.0085046 (PMC3877357; doi:10.1371/journal.pone.0085046)
Supplement: Figure S1 — rhSP-D does not affect viability of human PBMCs from healthy donors. (DOCX) [file pone.0085046.s001.docx]

**Figure S1**

**
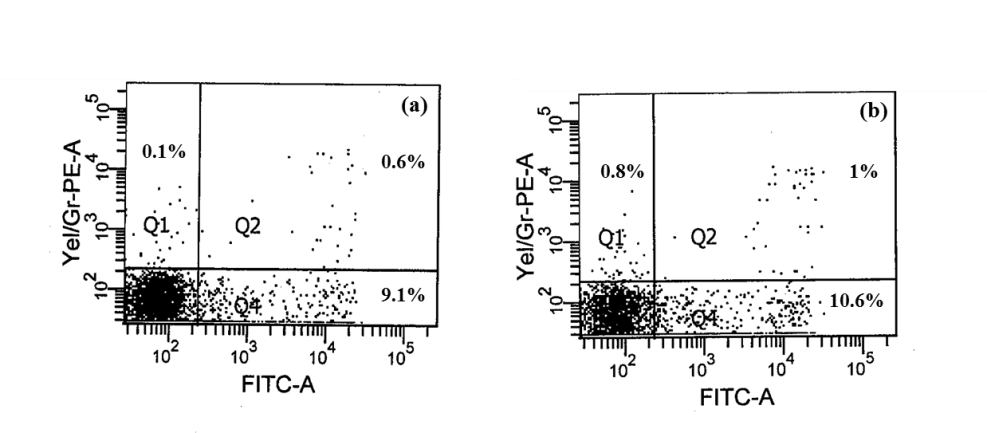
**

**Figure S1. rhSP-D does not affect viability of human PBMCs from healthy donors.**

Human PBMCs from healthy donors were incubated with or without rhSP-D at a concentration of 20µg/ml. Apoptosis was assessed by Annexin V-FITC staining using flow cytometry. (a) Untreated, (b) rhSP-D (20µg/ml) treated. The figure shows representative histograms from one of the three independent experiments with rhSP-D.
