# Supplementary material for: Human Surfactant Protein D Alters Oxidative Stress and HMGA1 Expression to Induce p53 Apoptotic Pathway in Eosinophil Leukemic Cell Line
Source: PLoS One. 2013 Dec 31;8(12):e85046. doi: 10.1371/journal.pone.0085046 (PMC3877357; doi:10.1371/journal.pone.0085046)
Supplement: Table S1 — Identification of differentially expressed proteins of AML14.3D10 cell line on treatment with rhSP-D by MALDI-TOF and/or MALDI-TOF-MS/MS analysis. (DOCX) [file pone.0085046.s002.docx]

**Table S1. Identification of differentially expressed proteins of AML14.3D10 cell line on treatment with rhSP-D by MALDI-TOF and/or MALDI-TOF-MS/MS analysis.**

| **Spot No.** | **Locus Tag** | **Protein name** | **Protein Status** | **Theoretical Mass (Da)/pI** | **Experimental Mass (Da)/pI** | **MS data** | | | **MS/MS data** | | | | |
| --- | --- | --- | --- | --- | --- | --- | --- | --- | --- | --- | --- | --- | --- |
|  |  |  |  |  |  | **No. of peptides matched** | **% sequence coverage** | **Mascot Score** | **No. of peptides matched** | **Total Score** | **Peptides matched** | **Peptide Span** | **Peptide Score** |
| 1 | gi\|71042034 | Chain A, X-Ray Crystal Structure Of A Chemically Synthesized Ubiquitin | Decreased | 85.4/6.8 | 10.8/7.6 | 6 | 77 | 75 | 3 | 128 | TITLEVEPSDTIENVK | 12 - 27 | 36 |
|  |  |  |  |  |  |  |  |  |  |  | IQDKEGIPPDQQR | 30 – 42 | 81 |
| 2 | gi\|1633186 | Chain A, Pkci-1-Apo+zinc | Decreased | 13.9/6.2 | 14.4/7.2 | 10 | 87 | 139 | 4 | 85 | AQVARPGGDTIFGK | 8 - 21 | 30 |
| 3 | gi\|14277700 | Ribosomal protein S12 | Decreased | 14.9/6.8 | 17.0/7.2 | 8 | 63 | 81 | 5 | 175 | LGEWVGLCK | 85 – 93 | 52 |
|  |  |  |  |  |  |  |  |  |  |  | ESQAKDVIEEYFK | 117 – 129 | 61 |
|  |  |  |  |  |  |  |  |  |  |  | DVIEEYFK | 122 – 129 | 52 |
| 4 | gi\|4557581 | Fatty acid binding protein 5 (psoriasis-associated) | Decreased | 15.5/6.6 | 14.6/6.9 | 11 | 69 | 104 | 3 | 110 | TTQFSCTLGEKFEETTADGR | 62 - 81 | 91 |
| 5 | gi\|12408675 | Prefoldin subunit 2 | Absent | 16.7/6.2 | 18.5/6.8 | 12 | 51 | 113 | 1 | 79 | GAVSAEQVIAGFNR | 19 - 32 | 79 |
| 6 | gi\|113419208 | PREDICTED: similar to Putative S100 calcium-binding protein A11 pseudogene | Absent | 11.4/9.0 | 11.0/6.6 | 4 | 40 | 67 |  |  |  |  |  |
| 7 | gi\|83754516 | Chain B, Crystal Structure Of The Chip-Ubc13-Uev1a Complex | Decreased | 17.3/6.2 | 16.0/6.5 | 12 | 78 | 159 | 4 | 177 | LLAEPVPGIKAEPDESNAR | 15 – 33 | 46 |
|  |  |  |  |  |  |  |  |  |  |  | IYHPNVDKLGR | 75 – 85 | 58 |
|  |  |  |  |  |  |  |  |  |  |  | TNEAQAIETAR | 131 - 141 | 46 |
| 8 | gi\|4759212 | Tubulin-specific chaperone a | Decreased | 12.9/5 | 17.0/5.6 | 6 | 26 | 57 | 2 | 32 |  |  |  |
| 9 | gi\|8922762 | Membrane-type 1 matrix metalloproteinase cytoplasmic  tail binding protein-1 | Decreased | 21.5/5.4 | 19.1/6.1 | 9 | 60 | 83 |  |  |  |  |  |
| 10 | gi\|5031593 | Actin related protein 2/3 complex subunit 5 | Decreased | 16.4/5.5 | 18.4/6.1 | 8 | 60 | 83 | 2 | 93 | ALAAGGVGSIVR | 132-143 | 51 |
|  |  |  |  |  |  |  |  |  |  |  | QGNMTAALQAALKNPPINTK | 48-67 | 42 |
| 11 | gi\|13124881 | Core-binding factor, beta subunit isoform 1 | Absent | 22.2/5.5 | 22.0/6.2 | 13 | 56 | 127 |  |  |  |  |  |
| 12 | gi\|5031635 | Cofilin 1 (non-muscle) | Decreased | 18.7/8.2 | 18.6/7.0 | 11 | 69 | 118 | 5 | 187 | YALYDATYETK | 82 – 92 | 75 |
|  |  |  |  |  |  |  |  |  |  |  | HELQANCYEEVKDR | 133 - 146 | 35 |
| 13 | gi\|32483377 | Peroxiredoxin 3 isoform b | Decreased | 26.1/7.0 | 25.3/6.7 | 8 | 50 | 98 | 3 | 126 | DYGVLLEGSGLALR | 171–184 | 38 |
|  |  |  |  |  |  |  |  |  |  |  | HLSVNDLPVGR | 197- 207 | 62 |
| 14 | gi\|7546411 | Chain A, X-Ray Crystal Structure For Human  Manganese Superoxide Dismutase, Q143a | Decreased | 22.2/6.9 | 21.6/7.8 | 14 | 82 | 181 |  |  | NVRPDYLK | 171-178 | 48 |
|  |  |  |  |  |  |  |  |  |  |  | GELLEAIKR | 91-99 | 48 |
|  |  |  |  |  |  |  |  |  |  |  | DFGSFDKFK | 100-108 | 48 |
|  |  |  |  |  |  |  |  |  |  |  | GDVTAQIALQPALK | 52-65 | 61 |
|  |  |  |  |  |  |  |  |  |  |  | AIWNVINWENVTER | 179-192 | 32 |
| 15 | gi\|20070130 | Basic transcription factor 3 isoform B | Decreased | 17.7/6.9 | 22.0/7.9 | 11/50 | 63 | 108 |  |  |  |  |  |
| 16 | gi\|999892 | Chain A, Triosephosphate Isomerase (Tim) (E.C.5.3.1.1) Complexed With 2-Phosphoglycolic Acid | Decreased | 26.8/6.5 | 32.8/7.5 | 22 | 91 | 305 | 5 | 298 | KFFVGGNWK | 5–13 | 53 |
|  |  |  |  |  |  |  |  |  |  |  | VPADTEVVCAPPTAYIDFAR | 33–52 | 65 |
|  |  |  |  |  |  |  |  |  |  |  | IAVAAQNCYK | 59–68 | 97 |
|  |  |  |  |  |  |  |  |  |  |  | DCGATWVVLGHSER | 85–98 | 64 |
| 17 | gi\|7706322 | Homeobox prox 1 | Absent | 28.2/6.2 | 32.9/7.0 | 18/50 | 70 | 204 | 2 | 44 | NAEPLINLDVNNPDFK | 116-131 | 36 |
| 18 | gi\|45768728 | Ubiquinol-cytochrome c reductase, Rieske iron-sulfur  polypeptide 1 | Decreased | 29.9/8.6 | 25.3/7.2 | 14/50 | 49 | 154 | 2 | 72 | GKPLFVR | 171-177 | 37 |
|  |  |  |  |  |  |  |  |  |  |  | VPDFSEYR | 85-92 | 35 |
| 19 | gi\|4505587 | Platelet-activating factor acetylhydrolase, isoform Ib,  gamma subunit 29kDa | Decreased | 25.8/6.3 | 23.9/7.2 | 15/50 | 73 | 183 | 4 | 199 | VVVLGLLPR | 133-141 | 45 |
|  |  |  |  |  |  |  |  |  |  |  | AIVQLVNER | 119-127 | 50 |
|  |  |  |  |  |  |  |  |  |  |  | LENGELEHIRPK | 84-95 | 64 |
|  |  |  |  |  |  |  |  |  |  |  | AIVQLVNERQPQAR | 119-132 | 39 |
| 20 | gi\|123377 | High-mobility group I  (HMGA1a) | Decreased | 11.7/10.8 | 28.1/7.0 | 12 | 71 | 111 |  |  |  |  |  |
| 21 | gi\|123377 | High-mobility group I  (HMGA1a) | Decreased | 11.7/10.8 | 28.0/6.8 | 13 | 57 | 104 |  |  |  |  |  |
| 22 | gi\|11056044 | Pyrophosphatase 1 | Increased | 33.1/5.5 | 39.7/6.1 | 18 | 78 | 202 | 5 | 154 | AAPFSLEYR | 10 – 18 | 36 |
|  |  |  |  |  |  |  |  |  |  |  | YVANLFPYK | 80 – 88 | 47 |
|  |  |  |  |  |  |  |  |  |  |  | LKPGYLEATVDWFR | 178 - 191 | 38 |
| 23 | gi\|387010 | Pyruvate dehydrogenase E1-beta subunit precursor | Increased | 36.8/5.4 | 38.4/6.0 | 16 | 57 | 148 | 3 | 47 | IMEGPAFNFLDAPAVR | 309 – 324 | 40 |
| 24 | gi\|5453597 | F-actin capping protein alpha-1 subunit | Increased | 33.1/5.4 | 39.3/6.0 | 12 | 52 | 148 | 4 | 202 | FITHAPPGEFNEVFNDVR | 20 – 37 | 71 |
|  |  |  |  |  |  |  |  |  |  |  | LLLNNDNLLR | 38 – 47 | 59 |
|  |  |  |  |  |  |  |  |  |  |  | TIDGQQTIIACIESHQFQPK | 147 - 166 | 45 |
| 25 | gi\|113422970 | PREDICTED: similar to Copper chaperone for SOD | Increased | 27.8/5.8 | 38.4/5.9 | 6/23 | 22 | 67 |  |  |  |  |  |
| 26 | gi\|63253298 | Spermidine synthase | Increased | 34.4/5.3 | 38.4/5.7 | 6/25 | 11 | 60 | 2 | 33 | VLIIGGGDGGVLR | 97 - 109 | 177 |
| 27 | gi\|72534660 | Splicing factor, arginine/serine-rich 7 | Increased | 27.6/11.8 | 38.7/5.5 | 13/50 | 42 | 111 | 4 | 145 | AFSYYGPLR | 30-39 | 57 |
| 28 | gi\|736704 | Cytoskeleton associated protein | Increased | 21.9/4.8 | 38.1/5.4 | 5/18 | 34 | 62 |  |  | VYVGNLGTGAGKGELER | 13-29 | 45 |
| 29 | gi\|40805827 | Epsilon subunit of coatomer protein complex isoform c | Increased | 28.9/5.1 | 39.5/5.3 | 9/26 | 38 | 114 |  |  |  |  |  |
| 30 | gi\|12654549 | NOP17 | Increased | 32.6/5.0 | 39.8/5.3 | 5 | 22 | 58 | 1 | 41 | IQELGDLYTPAPGR | 187 – 200 |  |
| 31 | gi\|56966800 | Chain A, Solution Structure Of Rrm Domain In Hnrpc | Increased | 10.3/9.7 | 41.7/5.2 | 8/40 | 73 | 113 |  |  |  |  |  |
| 32 | gi\|62088106 | Thioredoxin-like 1 | Increased | 62.3/5.1 | 41.7/5.3 | 15 | 28 | 102 | 3 | 69 | VGVKPVGSDPDFQPELSGAGSR | 2 – 23 | 45 |
| 33 | gi\|7657649 | Tropomodulin 3 | Increased | 39.7/5.1 | 46.3/5.6 | 6 | 23 | 57 | 2 | 80 | ILPVFDEPPNPTNVEESLKR | 173-192 | 45 |
|  |  |  |  |  |  |  |  |  |  |  | FGYQFTQQGPR | 318-328 | 36 |
| 34 | gi\|20149635 | p47 protein isoform a | Increased | 40.5/5.0 | 50.3/5.4 | 14 | 45 | 165 | 6 | 166 | SGQQIVGPPR | 102 – 111 | 44 |
|  |  |  |  |  |  |  |  |  |  |  | SGFSLDNGELR | 189 – 199 | 34 |
|  |  |  |  |  |  |  |  |  |  |  | EANLLNAVIVQR | 357 – 368 | 32 |
| 35 | gi\|7106439 | Tubulin, beta 5 | Increased | 50.1/4.8 | 53.0/5.2 | 28 | 59 | 252 | 2 | 66 | FPGQLNADLR | 242 – 251 | 32 |
|  |  |  |  |  |  |  |  |  |  |  | YLTVAAVFR | 310 – 318 | 34 |
| 36 | gi\|7106439 | Tubulin, beta 5 | Increased | 50.1/4.8 | 53.0/5.3 | 21/50 | 50 | 192 | 6 | 164 | FPGQLNADLR | 242 – 251 | 52 |
|  |  |  |  |  |  |  |  |  |  |  | YLTVAAVFR | 310 – 318 | 30 |
|  |  |  |  |  |  |  |  |  |  |  | TAVCDIPPR | 351 – 359 | 41 |
| 37 | gi\|338695 | Tubulin, beta | Increased | 50.2/4.8 | 53.0/5.4 | 26/50 | 55 | 272 | 3 | 54 | YLTVAAVFR | 310 – 318 | 31 |
| 38 | gi\|15277503 | ACTB protein | Increased | 40.5/5.5 | 46.3/5.7 | 18 | 50 | 161 | 6 | 254 | AGFAGDDAPR | 19 – 28 | 41 |
|  |  |  |  |  |  |  |  |  |  |  | VAPEEHPVLLTEAPLNPK | 96 – 113 | 47 |
|  |  |  |  |  |  |  |  |  |  |  | GYSFTTTAER | 239 – 254 | 77 |
|  |  |  |  |  |  |  |  |  |  |  | QEYDESGPSIVHR | 360 – 372 | 61 |
| 39 | gi\|15277503 | ACTB protein | Increased | 40.5/44.3 | 44.3/5.8 | 18 | 50 | 142 | 5 | 187 | AGFAGDDAPR | 19 – 28 | 44 |
|  |  |  |  |  |  |  |  |  |  |  | GYSFTTTAER | 197 – 206 | 38 |
|  |  |  |  |  |  |  |  |  |  |  | SYELPDGQVITIGNER | 239 – 254 | 77 |
| 40 | gi\|5729991 | Proteasome 26S ATPase subunit 4 isoform 1 | Increased | 47.5/5.1 | 53.9/5.5 | 21 | 60 | 224 | 5 | 115 | EAVELPLTHFELYK | 179 – 192 | 56 |
| 41 | gi\|89574029 | Mitochondrial ATP synthase, H+ transporting F1 complex beta subunit | Increased | 48.1/5.0 | 58.5/5.4 | 30 | 69 | 285 | 5 | 290 | AHGGYSVFAGVGER | 236-249 | 97 |
|  |  |  |  |  |  |  |  |  |  |  | LVLEVAQHLGESTVR | 105-119 | 59 |
|  |  |  |  |  |  |  |  |  |  |  | VRLDSGAPIKIPVGPETLGR | 135-153 | 69 |
|  |  |  |  |  |  |  |  |  |  |  | AIAELGIYPAVDPLDSTSR | 398-416 | 41 |
| 42 | gi\|109106425 | PREDICTED: proteasome 26S ATPase subunit 3 isoform 2 | Increased | 45.5/5.4 | 58.5/5.5 | 17 | 42 | 154 |  |  |  |  |  |
| 43 | gi\|3095186 | Cargo selection protein TIP47 | Increased | 47.2/5.3 | 57.1/5.5 | 10 | 34 | 99 |  |  |  |  |  |
| 44 | gi\|4826760 | Heterogeneous nuclear ribonucleoprotein F | Increased | 46.0/5.4 | 57.9/5.7 | 7 | 23 | 65 | 3 | 80 | VHIEIGPDGR | 317-326 | 35 |
| 45 | gi\|42794775 | Thioredoxin domain containing 5 isoform 2 | Increased | 44.4/5.8 | 55.8/5.9 | 22 | 58 | 271 |  |  |  |  |  |
| 46 | gi\|30089997 | Actin-like 6A isoform 2 | Increased | 43.7/5.8 | 55.9/6.0 | 13 | 34 | 95 |  |  |  |  |  |
| 47 | gi\|386758 | GRP78 precursor | Increased | 72.2/5.0 | 73.8/5.2 | 24 | 41 | 211 | 6 | 170 | VTHAVVTVPAYFNDAQR | 165– 181 | 36 |
|  |  |  |  |  |  |  |  |  |  |  | IINEPTAAAIAYGLDKR | 198 – 214 | 36 |
|  |  |  |  |  |  |  |  |  |  |  | IEIESFYEGEDFSETLTR | 306 – 323 | 35 |
| 48 | gi\|6470150 | GRP78 precursor | Increased | 71.0/5.2 | 73.8/5.3 | 29 | 48 | 292 | 7 | 457 | ITPSYVAFTPEGER | 61 – 74 | 33 |
|  |  |  |  |  |  |  |  |  |  |  | VTHAVVTVPAYFNDAQR | 165 – 181 | 72 |
|  |  |  |  |  |  |  |  |  |  |  | IINEPTAAAIAYGLDKR | 198 – 214 | 65 |
|  |  |  |  |  |  |  |  |  |  |  | IEIESFYEGEDFSETLTR | 306 – 323 | 111 |
|  |  |  |  |  |  |  |  |  |  |  | AKFEELNMDLFR | 324 – 335 | 73 |
|  |  |  |  |  |  |  |  |  |  |  | DNHLLGTFDLTGIPPAPR | 474 - 491 | 86 |
| 49 | gi\|6470150 | GRP78 precursor | Increased | 71.0/5.2 | 73.8/5.4 | 26/50 | 43 | 246 | 6 | 322 | VTHAVVTVPAYFNDAQR | 165 – 181 | 72 |
|  |  |  |  |  |  |  |  |  |  |  | IINEPTAAAIAYGLDKR | 198 – 214 | 59 |
|  |  |  |  |  |  |  |  |  |  |  | IEIESFYEGEDFSETLTR | 306 – 323 | 74 |
|  |  |  |  |  |  |  |  |  |  |  | AKFEELNMDLFR | 324 – 335 | 60 |
|  |  |  |  |  |  |  |  |  |  |  | AKFEELNMDLFR | 324 – 335 | 33 |
|  |  |  |  |  |  |  |  |  |  |  | DNHLLGTFDLTGIPPAPR | 474 - 491 | 57 |
| 50 | gi\|62897681 | Calreticulin precursor variant | Increased | 47.1/4.3 | 65.6/5.5 | 8/31 | 23 | 74 | 3 | 150 | VHVIFNYK | 144-151 | 56 |
|  |  |  |  |  |  |  |  |  |  |  | NVLINKDIR | 154-162 | 34 |
|  |  |  |  |  |  |  |  |  |  |  | KVHVIFNYK | 143-151 | 60 |
| 51 | gi\|55958547 | Heterogeneous nuclear ribonucleoprotein K | Increased | 42.0/5.4 | 63.3/5.6 | 12/39 | 35 | 108 | 2 | 57 | GSDFDCELR | 140-148 | 33 |
| 52 | gi\|31542947 | Chaperonin | Increased | 61.2/5.7 | 62.6/5.7 | 29/50 | 54 | 250 | 7 | 368 | TVIIEQSWGSPK | 61 – 72 | 93 |
|  |  |  |  |  |  |  |  |  |  |  | TLNDELEIIEGMKFDR | 206 – 221 | 31 |
|  |  |  |  |  |  |  |  |  |  |  | KPLVIIAEDVDGEALSTLVLNR | 269 – 290 | 123 |
|  |  |  |  |  |  |  |  |  |  |  | AAVEEGIVLGGGCALLR | 430 – 446 | 66 |
|  |  |  |  |  |  |  |  |  |  |  | IGIEIIKR | 463 - 470 | 37 |
| 53 | gi\|55958544 | Heterogeneous nuclear ribonucleoprotein K  transformation upregulated nuclear protein | Increased | 47.8/5.5 | 63.7/5.8 | 19/33 | 40 | 223 | 3 | 107 | GSDFDCELR | 140 - 148 | 33 |
|  |  |  |  |  |  |  |  |  |  |  | DYDDMSPR | 279 – 286 | 31 |
|  |  |  |  |  |  |  |  |  |  |  | NLPLPPPPPPR | 306 - 316 | 43 |
| 54 | gi\|24234688 | Heat shock 70kDa protein 9B precursor | Increased | 74.0/5.9 | 71.0/5.9 | 10/33 | 17 | 76 |  |  |  |  |  |
| 55 | gi\|21040386 | HSPA9 protein | Increased | 74.0/6.0 | 65.6/6.1 | 30/50 | 46 | 278 | 4 | 275 | VQQTVQDLFGR | 395-405 | 71 |
|  |  |  |  |  |  |  |  |  |  |  | AQFEGIVTDLIR | 349-360 | 83 |
|  |  |  |  |  |  |  |  |  |  |  | LLGQFTLIGIPPAPR | 499-513 | 55 |
|  |  |  |  |  |  |  |  |  |  |  | NAVITVPAYFNDSQR | 188-202 | 66 |
| 56 | gi\|62088704 | Heterogeneous nuclear ribonucleoprotein K isoform a  variant | Increased | 49.0/5.5 | 62.9/6.1 | 15/33 | 37 | 162 | 2 | 102 | DYDDMSPR | 255-262 | 38 |
| 57 | gi\|4504169 | Glutathione synthetase | Increased | 52.5/5.7 | 57.4/6.2 | 20/36 | 46 | 233 |  |  |  |  |  |
| 58 | gi\|58082085 | Aminoacylase 1-like 2 | Increased | 48.1/5.6 | 54.9/6.1 | 8/25 | 17 | 65 | 2 | 61 | GALEGLPRPPPPVK | 90-103 | 39 |
| 59 | gi\|58082085 | Aminoacylase 1-like 2 | Increased | 48.1/5.6 | 54.9/6.2 | 15/50 | 44 | 132 | 3 | 119 | VLTHFFER | 61-68 | 48 |
|  |  |  |  |  |  |  |  |  |  |  | GALEGLPRPPPPVK | 142-155 | 56 |
| 60 | gi\|118084547 | Aryl hydrocarbon receptor interacting protein | Increased | 38.1/5.8 | 40.6/7.0 | 8/17 | 26 | 106 |  |  |  |  |  |
| 61 | gi\|16307182 | TALDO1 protein | Increased | 35.5/9.1 | 40.2/6.9 | 6/20 | 23 | 62 |  |  |  |  |  |
| 62 | gi\|16307182 | TALDO1 protein | Increased | 35.5/9.1 | 39.8/6.5 | 7/24 | 26 | 69 |  |  |  |  |  |
| 63 | gi\|10241724 | Hypothetical protein | Increased | 31.8/5.8 | 39.9/6.4 | 8/33 | 30 | 79 |  |  |  |  |  |
| 64 | gi\|4506667 | Ribosomal protein P0 | Increased | 34.4/5.7 | 40.0/6.3 | 22/48 | 62 | 297 | 6 | 279 | AGAIAPCEVTVPAQNTGLGPEK | 113 – 134 | 55 |
|  |  |  |  |  |  |  |  |  |  |  | TSFFQALGITTK | 135 – 146 | 51 |
|  |  |  |  |  |  |  |  |  |  |  | GTIEILSDVQLIK | 150 – 162 | 76 |
|  |  |  |  |  |  |  |  |  |  |  | VLALSVETDYTFPLAEK | 248 - 264 | 58 |
| 65 | gi\|4504865 | KH-type splicing regulatory protein (FUSE binding protein 2) | Decreased | 73.5/6.8 | 75.5/7.6 | 23/50 | 45 | 215 | 6 | 179 | IGGGIDVPVPR | 321 – 331 | 35 |
|  |  |  |  |  |  |  |  |  |  |  | IINDLLQSLR | 385 – 394 | 69 |
|  |  |  |  |  |  |  |  |  |  |  | AINQQTGAFVEISR | 449 - 462 | 42 |
| 66 | gi\|54648253 | KHSRP protein | Decreased | 73.3/8.0 | 76.0/7.4 | 16/36 | 35 | 160 | 5 | 88 | IINDLLQSLR | 385 – 394 | 34 |
| 67 | gi\|4504035 | Guanine monophosphate synthetase | Decreased | 77.4/6.4 | 76.0/7.3 | 14/33 | 33 | 144 |  |  |  |  |  |
| 68 | gi\|17402900 | Far upstream element-binding protein | Decreased | 67.7/7.2 | 70.5/7.4 | 9/23 | 16 | 82 | 4 | 77 | IGGNEGIDVPIPR | 122-134 | 34 |
| 69 | gi\|619775 | This CDS feature is included to show the translation of the corresponding V_region. | Decreased | 11.3/9.4 | 68.0/7.3 | 5/37 | 63 | 58 |  |  |  |  |  |
| 70 | gi\|33874520 | SYNCRIP protein | Increased | 47.0/5.8 | 68.1/7.1 | 12/39 | 29 | 99 |  |  |  |  |  |
| 71 | gi\|112491363 | Chain A, Crystal Structure Of The Alpha Subunit Of  Human S- Adenosylmethionine Synthetase 2 | Increased | 44.1/6.0 | 76.8/6.9 | 11/40 | 24 | 85 | 3 | 67 | FVIGGPQGDAGLTGR | 250-264 | 36 |
| 72 | gi\|5174529 | Methionine adenosyltransferase II, alpha | Increased | 44.1/6.0 | 56.5/6.8 | 12/33 | 25 | 114 |  |  |  |  |  |
| 73 | gi\|5031753 | Heterogeneous nuclear ribonucleoprotein H1 | Increased | 49.5/5.9 | 58.1/6.7 | 22/50 | 53 | 199 | 4 | 154 | VHIEIGPDGR | 317-326 | 35 |
|  |  |  |  |  |  |  |  |  |  |  | HTGPNSPDTANDGFVR | 99-114 | 59 |
|  |  |  |  |  |  |  |  |  |  |  | ATENDIYNFFSPLNPVR | 300-316 | 32 |
| 74 | gi\|4505587 | Platelet-activating factor acetylhydrolase, isoform Ib,  gamma subunit 29kDa | Decreased | 25.8/6.3 | 58.5/6.8 | 14/43 | 63 | 175 | 5 | 236 | VVVLGLLPR | 133-141 | 48 |
|  |  |  |  |  |  |  |  |  |  |  | AIVQLVNER | 119-127 | 54 |
|  |  |  |  |  |  |  |  |  |  |  | LENGELEHIRPK | 84-95 | 77 |
|  |  |  |  |  |  |  |  |  |  |  | AIVQLVNERQPQAR | 119-132 | 37 |
| 75 | gi\|38455391 | Ribonuclease HI, large subunit | Increased | 33.7/5.1 | 38.5/5.9 | 6/31 | 23 | 55 |  |  |  |  |  |

List of the proteins that showed altered expression (≥ 3-fold change) on rhSP-D (10µg/ml) treatment in AML14.3D10 cells. Spot number relates to the protein spots marked on the 2-DE gel images of proteins extracted from rhSP-D treated and untreated AML14.3D10 cells (Figure 3, main manuscript).
