# Supplementary material for: Human Surfactant Protein D Alters Oxidative Stress and HMGA1 Expression to Induce p53 Apoptotic Pathway in Eosinophil Leukemic Cell Line
Source: PLoS One. 2013 Dec 31;8(12):e85046. doi: 10.1371/journal.pone.0085046 (PMC3877357; doi:10.1371/journal.pone.0085046)
Supplement: Table S2 — Function and functional categories of differentially expressed proteins of AML14.3D10 cells on treatment with rhSP-D. (DOCX) [file pone.0085046.s003.docx]

**Table S2. Function and functional categories of differentially expressed proteins of AML14.3D10 cells on treatment with rhSP-D**

| **Spot No.** | **Protein Name** | **Functions** *[www.hprd.org](http://www.hprd.org)/ [www.uniprot.org](http://www.uniprot.org) | **Up or downregulation of protein** |
| --- | --- | --- | --- |
| **Chaperones and Heat shock proteins** | | | |
| 47 | GRP78 precursor | Chaperone activity, protein metabolism* | Increased |
| 48 | GRP78 precursor | Chaperone activity, protein metabolism* | Increased |
| 49 | GRP78 precursor | Chaperone activity, protein metabolism* | Increased |
| 52 | Chaperonin | Chaperone activity, protein metabolism* | Increased |
| 50 | Calreticulin precursor variant | Chaperone activity, protein metabolism* | Increased |
| 54 | Heat shock 70kDa protein 9B precursor | Heat-shock cognate protein* | Increased |
| 55 | HSPA9 protein | Heat-shock cognate protein* | Increased |
| 6 | PREDICTED: similar to Copper chaperone for  superoxide dismutase | The copper chaperone for superoxide dismutase (CCS) is an intracellular metallochaperone required for incorporation of copper into the essential antioxidant enzyme copper/zinc superoxide dismutase (SOD1) (1). | Increased |
| 8 | Tubulin-specific chaperone a | Tubulin cofactor A gene silencing in mammalian cells induces changes in microtubule cytoskeleton, cell cycle arrest and cell death and G1 cell cycle arrest (2). | Decreased |
| **Oxidoreductases** | | | |
| 57 | Glutathione synthetase | Ligase activity, metabolism activity* | Increased |
| 71 | Chain A, Crystal Structure Of The Alpha Subunit Of Human S- Adenosylmethionine Synthetase 2 | Required for the (S-Adenosylmethionine (SAM, AdoMet). SAM is the important methyl donor used for synthesis of nucleic acids, phospholipids, creatine, and polyamines and for methylation of many bioactive molecules and in oxidative stress of hyperoxia, a part of SAM generated is directed toward cysteine/GSH in the transsulfuration pathway (3). | Increased |
| 72 | Methionine adenosyltransferase II, alpha (MAT) | In oxidative stress of hyperoxia, MAT increases progressively and part of SAM generated is directed toward cysteine/GSH in the transsulfuration pathway (3). | Increased |
| 32 | Thioredoxin-like 1 variant | Oxidoreductases* | Increased |
| 41 | Mitochondrial ATP synthase, H+ transporting F1 complex beta subunit | [Oxidoreductase activity](http://godatabase.org/cgi-bin/go.cgi?query=GO:0016491&view=details&search_constraint=terms&depth=0): [Protein metabolism](http://godatabase.org/cgi-bin/go.cgi?query=GO:0019538&view=details&search_constraint=terms&depth=0)* | Increased |
| 45 | Thioredoxin domain containing 5 isoform 2 | Protein-disulfide isomerase. Its expression is induced by hypoxia and its role may be to protect hypoxic cells from apoptosis (4) . | Increased |
| 18 | Ubiquinol-cytochrome c reductase, Rieske iron-sulfur polypeptide 1 complex III of mitochondria | Reductase, Metabolism ; Catalytic activity; Energy pathways* | Almost absent |
| **Energy Metabolism pathways** | | | |
| 23 | Pyruvate dehydrogenase E1-beta subunit precursor | Energy Metabolism; Catalytic activity; Energy pathway* | Increased |
| **Ubiquitin proteasome pathways** | | | |
| 1 | Chain A, X-Ray Crystal Structure Of A Chemically Synthesized Ubiquitin | Involved in ubiquitin-specific protease activity (5). | Decreased |
| 7 | Chain B, Crystal Structure Of The Chip-Ubc13-Uev1a Complex | The protein is involved in chaperoned ubiquitylation: CHIP U box E3 ubiquitin ligase (5). | Decreased |
| 40 | Proteasome 26S ATPase subunit 4 isoform 1 | The proteasome cleaves peptides in an ATP and ubiquitin-dependent process in a non-lysosomal pathway (6). | Increased |
| 42 | PREDICTED: proteasome 26S ATPase subunit 3 isoform 2 | Protein metabolism: Ubiquitin specific protease activity* | Increased |
| **Metabolism enzymes** | | | |
| 9 | Membrane-type 1 matrix metalloproteinase cytoplasmic  tail binding protein-1 (MTCBP-1) | MTCBP-1 acts as an eukaryotic aci-reductone dioxygenase (ARD) in the methionine salvage pathway (7). | Decreased |
| 26 | Spermidine synthase | Spermidine synthase have been shown to be inducible during lectin induced lymphocyte activation, cell proliferation, such as liver regeneration and liver compensatory growth, hormone-induced growth of tissues (8, 9). | Increased |
| 57 | Glutathione synthetase | Ligase activity, metabolism activity* | Increased |
| 58 | Aminoacylase 1-like 2 | Glutamate carboxypeptidases* | Increased |
| 59 | Aminoacylase 1-like 2 | Glutamate carboxypeptidases* | Increased |
| 16 | Chain A, Triosephosphate Isomerase (Tim) complexed with 2-Phosphoglycolic Acid | Intramolecular Oxidoreductase/Catalytic activity* | Decreased |
| 61 | TALDO1 protein | Transaldolase is important for the balance of metabolites in the pentose-phosphate pathway*. | Increased |
| 62 | TALDO1 protein | -As above- | Increased |
| 67 | Guanine monophosphate synthetase (GMP synthetase) | Involved in the de novo synthesis of guanine nucleotides which are not only essential for DNA and RNA synthesis, but also provide GTP, which is involved in a number of cellular processes important for cell division*. | Decreased |
| 22 | Pyrophosphatase 1 | tRNA aminoacylation for protein translation* | Increased |
| 4 | Fatty acid binding protein 5 (psoriasis-associated) | High specificity for fatty acids. Highest affinity for C18 chain length. Decreasing the chain length or introducing double bonds reduces the affinity. May be involved in keratinocyte differentiation* | Decreased |
| **Cytoskeleton** | | | |
| 46 | Actin-like 6A isoform 2 | The actin-related proteins are involved in diverse cellular processes, including vesicular transport, spindle orientation, nuclear migration and chromatin remodeling*. | Increased |
| 10 | Actin related protein 2/3 complex subunit 5 | -As above- | Decreased |
| 24 | F-actin capping protein alpha-1 subunit | Actin cytoskeleton organization, actin filament capping, blood coagulation, cellular component movement, innate immune response, protein complex assembly*. | Increased |
| 33 | Tropomodulin 3 (ubiquitous) | Blocks the elongation and depolymerization of the actin filaments at the pointed end. The Tmod/TM complex contributes to the formation of the short actin protofilament, which in turn defines the geometry of the membrane skeleton *. | Increased |
| 35 | Tubulin, beta 5 | Overexpression of a mouse class V beta-tubulin cDNA in mammalian cells produces a strong, dose-dependent disruption of microtubule organization, increased microtubule fragmentation, and a concomitant reduction in cellular microtubule polymer levels. These changes also disrupt mitotic spindle assembly and block cell proliferation (10). | Increased |
| 36 | Tubulin, beta 5 | -As above- | Increased |
| 37 | Tubulin, beta | Microtubule regulation in mitosis: tubulin phosphorylation by the cyclin-dependent kinase Cdk1. As above (10). | Increased |
| 38 | ACTB protein | An ubiquitous protein involved in the formation of filaments that are a major component of the cytoskeleton. Interaction with myosin provides the basis of muscular contraction and many aspects of cell motility. | Increased |
| 34 | ACTB protein | -As above- | Increased |
| 12 | Cofilin 1 (non-muscle) | Cytoskeleton associated protein: cytoskeleton organization and bigenesis* | Decreased |
| **RNA binding and metabolism associated proteins** | | | |
| 73 | Heterogeneous nuclear ribonucleoprotein H1 | RNA binding protein; Regulation of nucleobase, nucleoside, nucleotide and nucleic acid metabolism* | Increased |
| 51 | Heterogeneous nuclear ribonucleoprotein K isoform a variant | RNA binding protein; Regulation of nucleobase, nucleoside, nucleotide and nucleic acid metabolism* | Increased |
| 53 | Heterogeneous nuclear ribonucleoprotein K transformation upregulated nuclear protein | -As above- | Increased |
| 56 | Heterogeneous nuclear ribonucleoprotein K | -As above- | Increased |
| 31 | Chain A, Solution Structure Of Rrm Domain in HNRPC | Single HNRPC tetramers bind 230-240 nucleotides. May play a role in the early steps of spliceosome assembly and pre-mRNA splicing (11). | Increased |
| 70 | SYNCRIP protein (hnRNP-Q, synaptogamin binding) | RNA binding protein; Regulation of nucleobase, nucleoside, nucleotide and nucleic acid metabolism NS1 associated protein * | Increased |
| 30 | NOP17 | Involved in pre-rRNA processing (12). | Increased |
| 27 | Splicing factor, arginine/serine-rich 7 | RNA binding protein; Regulation of nucleobase, nucleoside, nucleotide and nucleic acid metabolism * | Increased |
| 75 | Ribonuclease HI, large subunit | Rnase HI is the major enzyme and shows increased activity during DNA replication (13). | Increased |
| 65 | KH-type splicing regulatory protein | KH binds single-stranded RNA or DNA. It is found in a wide variety of proteins including ribosomal proteins, transcription factors and post-transcriptional modifiers of mRNA. mRNA export from nucleus; nuclear mRNA splicing, via spliceosome; regulation of transcription, DNA-dependent; RNA splicing*. | Decreased |
| 66 | KHSRP protein | -As above- | Decreased |
| 68 | Far upstream element-binding protein | This is a ssDNA binding protein that activates the far upstream element (FUSE) of c-myc and stimulates expression of c-myc in undifferentiated cells. This protein has been shown to function as an ATP-dependent DNA helicase (*14)*. | Decreased |
| **Transcription associated proteins** | | | |
| 15 | Basic transcription factor 3 isoform B | This protein forms a stable complex with RNA polymerase IIB and is required for transcriptional initiation*. | Decreased |
| 11 | Core-binding factor, beta subunit isoform 1 | The protein is the beta subunit of a heterodimeric core-binding transcription factor belonging to the PEBP2/CBF transcription factor family which master-regulates a host of genes specific to hematopoiesis (e.g., RUNX1) and osteogenesis (e.g., RUNX2) (15). | Absent |
| 17 | Homeobox prox 1 | Transcription regulatory protein :Transcription regulatory activity* | Absent |
| **Translation associated proteins** | | | |
| 3 | Ribosomal protein S12 | 40S ribosomal protein S12: This is a ribosomal protein and component of the 40S subunit. The protein belongs to the S12E family of ribosomal proteins. Increased expression of this gene in colorectal cancers compared to matched normal colonic mucosa has been observed (16, 17). | Decreased |
| 64 | Ribosomal protein P0 | A ribosomal protein that is a component of the 60S subunit. It is a neutral phosphoprotein with a C-terminal end that is nearly identical to the C-terminal ends of the acidic ribosomal phosphoproteins P1 and P2. The P0 protein can interact with P1 and P2 to form a pentameric complex consisting of P1 and P2 dimers, and a P0 monomer (17). | Decreased |
| 69 | This CDS feature is included to show the translation of the corresponding V_region |  | Decreased |
| **Inflammation and Survival proteins** | | | |
| 20 | High-mobility group box 1. Nuclear phosphoprotein HMGA1a | High-mobility group A1 (HMGA1) overexpression and gene rearrangement are frequent events in human cancer. HMGA1 overexpression promoted HIPK2 relocalization in the cytoplasm and inhibition of p53 apoptotic function (18). | Decreased |
| 21 | High-mobility group box 1 | -As above- | Decreased |
| 2 | Chain A, Pkci-1-Apo+zinc | Protein Kinase C Interacting (inhibitory). Protein PKCI and related proteins belong to the ubiquitous HIT family of hydrolases that act on alpha-phosphates of ribonucleotides. Modulates proteasomal degradation of target proteins by the SCF (SKP2-CUL1-F-box protein) E3 ubiquitin-protein ligase complex*. | Decreased |
| 19 | Platelet-activating factor acetylhydrolase, isoform Ib, gamma subunit 29kDa | Inactivates PAF by removing the acetyl group at the sn-2 position. This is a catalytic subunit*. | Decreased |
| 74 | Platelet-activating factor acetylhydrolase, isoform Ib, gamma subunit 29kDa | -As above- | Decreased |
| **Vesicle fusion, synthesis and trafficking** | | | |
| 29 | Epsilon subunit of coatomer protein complex isoform c | This is required for budding from Golgi membranes, and is essential for the retrograde Golgi-to-ER transport of dilysine-tagged proteins*. | Increased |
| 34 | p47 protein isoform a | p47 is an adaptor molecule of the cytosolic ATPase p97. The principal role of the p97-p47 complex is to regulate membrane fusion events (19). | Increased |
| 28 | Cytoskeleton associated protein | High-affinity epithelial cell surface receptor for APF. Mediates the anchoring of the endoplasmic reticulum to microtubules*. | Increased |
| **Others** | | | |
| 60 | Aryl hydrocarbon receptor interacting protein | May play a positive role in AHR-mediated (aromatic hydrocarbon receptor) signaling, possibly by influencing its receptivity for ligand and/or its nuclear targeting. Cellular negative regulator of the hepatitis B virus (HBV) X protein*. | Increased |
| 63 | Hypothetical protein |  | Increased |
| 6 | PREDICTED: similar to Putative S100 calcium-binding protein A11 pseudogene | The S-100 domain is a subfamily of the EF-hand CaBPs, expressed exclusively in vertebrates, and implicated in intracellular and extracellular regulatory activities expressed in leukocytes (20*).* | Absent |

**References:**

1. Rae TD, Schmidt PJ, Pufahl RA, Culotta VC, O'Halloran TV. (1999) Undetectable intracellular free copper: the requirement of a copper chaperone for superoxide dismutase. Science 284(5415):805-8.
2. Nolasco S, Bellido J, Gonçalves J, Zabala J, Soares H. (2005) Tubulin cofactor A gene silencing in mammalian cells induces changes in microtubule cytoskeleton, cell cycle arrest and cell death.  *FEBS Letters.* 579(17):3515.
3. Panayiotidis MI, Stabler SP, Ahmad A, Pappa A, Legros LH Jr, et al. (2006) Activation of a novel isoform of methionine adenosyl transferase 2A and increased S-adenosylmethionine turnover in lung epithelial cells exposed to hyperoxia. Free Radic Biol Med 15;40(2):348-58.
4. Jacquier-Sarlin MR, Polla BS. (1996) Dual regulation of heat-shock transcription factor (HSF) activation and DNA-binding activity by H_2_O_2_: role of thioredoxin. Biochem J 318 (Pt 1):187.
5. Qian, SB, McDonough H, Boellmann F, Cyr DM, Patterson C. (2006) CHIP-mediated stress recovery by sequential ubiquitination of substrates and Hsp70. Nature 440(7083):551.
6. Coux O, Tanaka K, Goldberg AL. (1996) Structure and functions of the 20S and 26S proteasomes. Annu Rev Biochem 65:801.
7. Hirano W, Gotoh I, Uekita T, Seiki M. (2005) Membrane-type 1 matrix metalloproteinase cytoplasmic tail binding protein-1 (MTCBP-1) acts as an eukaryotic aci-reductone dioxygenase (ARD) in the methionine salvage pathway. Genes Cells 10(6):565.
8. Nishikawa Y, Kar S, Wiest L, Pegg AE, Carr BI. (1997) Inhibition of spermidine synthase gene expression by transforming growth factor-β1 in hepatoma cells. Biochem J 321:537.
9. Pignatti C, Tantini B, Stefanelli C, Flamigni F. (2004) Signal transduction pathways linking polyamines to apoptosis. Amino Acids 27:359.
10. Bhattacharya R, Cabral F. (2004) A ubiquitous beta-tubulin disrupts microtubule assembly and inhibits cell proliferation. Mol Biol Cell 15(7):3123.
11. Séraphin B, Abovich N, Rosbash M. (1991) Genetic depletion indicates a late role for U5 snRNP during in vitro spliceosome assembly. Nucleic Acids Res 25;19(14):3857-60.
12. Gonzales FA, Zanchin NI, Luz JS, Oliveira CC. (2005) Characterization of Saccharomyces cerevisiae Nop17p, a novel Nop58p-interacting protein that is involved in Pre-rRNA processing. J Mol Bio 346(2):437.
13. Gaidamakov SA, Gorshkova II, Schuck P, Steinbach PJ, Yamada H, Crouch RJ, Cerritelli SM. (2005) Eukaryotic RNases H1 act processively by interactions through the duplex RNA-binding domain. Nucleic Acids Res 33(7):2166.
14. Duncan R, Bazar L, Michelotti G, Tomonaga T, Krutzsch H, et al. (1994) A sequence-specific, single-strand binding protein activates the far upstream element of c-myc and defines a new DNA-binding motif. Genes Dev 15;8(4):465-80.
15. Zhang DE, Hetherington CJ, Meyers S, Rhoades KL, Larson CJ, et al. 1996. CCAAT enhancer binding protein (C/EBP) and AML1 (CBF alpha2) synergistically activate the macrophage colony-stimulating factor receptor promoter. Mol Cell Biol 16:1231.
16. Pogue-Geile K, Geiser JR, Shu M, Miller C, Wool IG, et al. (1991) Ribosomal protein genes are overexpressed in colorectal cancer: isolation of a cDNA clone encoding the human S3 ribosomal protein. Mol Cell Biol 11(8):3842.
17. Barnard GF, Staniunas RJ, Bao S, Mafune K, Steele GD Jr, et al. (1992) Increased expression of human ribosomal phosphoprotein P0 messenger RNA in hepatocellular carcinoma and colon carcinoma. Cancer Res 52(11):3067.
18. Pierantoni GM, Rinaldo C, Mottolese M, Di Benedetto A, Esposito F, et al. (2007) High-mobility group A1 inhibits p53 by cytoplasmic relocalization of its proapoptotic activator HIPK2. J Clin Invest 117(3):693.
19. Yuan X, Simpson P, McKeown C, Kondo H, Uchiyama K, et al. (2004) Structure, dynamics and interactions of p47, a major adaptor of the AAA ATPase, p97. EMBO J 7;23(7):1463-73.
20. Donato R. (2003) Intracellular and extracellular roles of S100 proteins. Microsc Res Tech 15;60(6):540-51.
